# Cardiac Damage Staging Predicts Outcomes in Aortic Valve Stenosis After Aortic Valve Replacement: Meta-Analysis

**DOI:** 10.1016/j.jacadv.2024.100959

**Published:** 2024-05-22

**Authors:** Omar M. Abdelfattah, Xander Jacquemyn, Michel Pompeu Sá, Hani Jneid, Ibrahim Sultan, David J. Cohen, Linda D. Gillam, Lucy Aron, Marie-Annick Clavel, Philippe Pibarot, Jeroen J. Bax, Samir R. Kapadia, Martin Leon, Philippe Généreux

**Affiliations:** a Division of Cardiology, University of Texas Medical Branch, Galveston, Texas, USA; b Department of Cardiovascular Sciences, KU Leuven, Leuven, Belgium; c Heart and Vascular Institute, University of Pittsburgh Medical Center, Pittsburgh, Pennsylvania, USA; d Clinical Trials Center, Cardiovascular Research Foundation, New York, New York, USA; e DeMatteis Cardiovascular Institute, St. Francis Hospital and Heart Center, Roslyn, New York, USA; f Gagnon Cardiovascular Institute, Morristown Medical Center, Morristown, New Jersey, USA; g Quebec Heart & Lung Institute, Laval University, Quebec City, Quebec, Canada; h Department of Cardiology, Leiden University Medical Center, Leiden, Netherlands; i Heart, Thoracic and Vascular Institute, Cleveland Clinic, Cleveland, Ohio, USA; j NewYork-Presbyterian Hospital/Columbia University, Medical Center, New York, New York, USA

**Keywords:** aortic stenosis, aortic valve replacement, cardiac damage, transcatheter aortic valve replacement

## Abstract

**Background:**

The prognostic value of cardiac damage staging classification based on the extent of extravalvular damage has been proposed in moderate/severe aortic stenosis (AS).

**Objectives:**

The purpose of this study was to assess the association of cardiac damage staging with mortality across the spectrum of patients with AS following aortic surgical or transcatheter aortic valve replacement (AVR).

**Methods:**

We conducted a pooled meta-analysis of Kaplan-Meier-derived reconstructed time-to-event data from studies published through February 2023.

**Results:**

In total, 16 studies (n = 14,499) met our eligibility criteria and included 12,282 patients with symptomatic severe AS and 2,217 patients with asymptomatic severe/moderate AS. For patients with symptomatic severe AS, all-cause mortality was 24.0%, 27.7%, 38.0%, 56.3%, and 57.3% at 5 years in patients with cardiac damage stage 0, 1, 2, 3, and 4, respectively (stage 0 as reference; HR in stage 1: 1.30 [95% CI: 1.03-1.64]; *P* = 0.029; stage 2: 1.74 [95% CI: 1.41-2.16]; *P* < 0.001; stage 3: 2.92 [95% CI: 2.35-3.64]; *P* < 0.001, and stage 4: 3.51 [95% CI: 2.79-4.41]; *P* < 0.001). For patients with asymptomatic moderate/severe AS, all-cause mortality was 19.3%, 36.9%, 51.7%, and 67.8% at 8 years in patients with cardiac damage stage 0, 1, 2, and 3 to 4, respectively (HR in stage 1: 1.70 [95% CI: 1.21-2.38]; *P* = 0.002; stage 2: 2.20 [95% CI: 1.60-3.02]; *P* < 0.001; and stage 3 to 4: 3.90 [95% CI: 2.79-5.47]; *P* < 0.001).

**Conclusions:**

In patients undergoing AVR across the symptomatic and severity spectrum of AS, cardiac damage staging at baseline has important prognostic implications. This pooled meta-analysis in patients undergoing AVR suggests that staging of baseline cardiac damage could be considered for timing and selection of therapy in patients with moderate or severe AS to determine the need for earlier AVR or adjunctive pharmacotherapy to prevent irreversible cardiac damage and improve the long-term prognosis.

## Central Illustration

**Figure:**
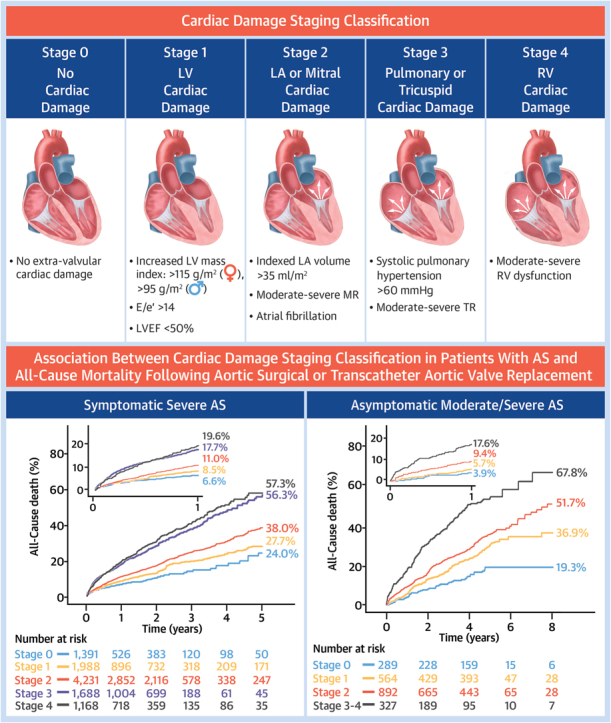

The current 2020 American College of Cardiology/American Heart Association and the 2021 European Society of Cardiology/European Association for Cardiothoracic Surgery guidelines recommend aortic valve replacement (AVR) for patients with aortic stenosis (AS) presenting with either severe AS with symptoms or low left ventricular (LV) ejection fraction (<50%) or when moderate AS co-exists with another indication for open heart surgery (ie, coronary artery bypass, other valve disease, aortopathy).^1^^,^^2^ Recently, a cardiac staging classification based on the extent of cardiac damage has been developed to stratify AS patients either before or after AVR.^3^, ^4^, ^5^ This novel staging of cardiac damage has been validated in multiple cohorts for AS, including moderate and severe symptomatic and asymptomatic patients.^3^^,^^6^, ^7^, ^8^, ^9^, ^10^, ^11^, ^12^, ^13^, ^14^, ^15^, ^16^, ^17^, ^18^, ^19^, ^20^, ^21^, ^22^, ^23^ Moreover, this classification has been validated in other valvular heart diseases including mitral regurgitation,^24^, ^25^, ^26^, ^27^, ^28^ tricuspid regurgitation,^29^^,^^30^ and aortic regurgitation.^31^

The extravalvular cardiac damage staging proposed by Généreux et al^3^ introduced a more holistic and multiparametric approach to assessing the various clinical and structural changes associated with AS. Such an approach of staging patients based on global cardiac health has shown prognostic utility in risk stratification and potentially in determining the optimal timing for AVR. Despite previous reports demonstrating the impact of cardiac damage staging in AS, some cardiac damage stages were under-represented given the considerably low number of patients in individual studies, making it somewhat challenging to elicit more robust conclusions. This pooled meta-analysis of Kaplan-Meier-derived reconstructed time-to-event data aims to assess the impact of cardiac damage staging across the spectrum of severity and symptoms of AS on all-cause and cardiovascular (CV) mortality after AVR.

## Methods

### Search strategy

This meta-analysis of observational studies^6^, ^7^, ^8^, ^9^, ^10^^,^^14^, ^15^, ^16^^,^^18^, ^19^, ^20^, ^21^, ^22^^,^^32^ and randomized trials^4^^,^^13^ was conducted to assess the long-term prognostic performance of cardiac damage staging classification across the spectrum of AS (ie, symptomatic severe, asymptomatic severe, and moderate AS) undergoing AVR. Data collection and reporting were performed in accordance with the Preferred Reporting Items for Systematic Reviews and Meta-Analyses guidelines and the Meta-analysis of Observational Studies in Epidemiology reporting guidelines.^33^^,^^34^ The study protocol was registered in the International Prospective Register of Systematic Reviews (PROSPERO) (CRD42023406744). Ethical approval was not required for this study-level meta-analysis.

Multiple electronic databases were systematically searched for all articles published by February 15, 2023, using the key terms “aortic valve stenosis,” “cardiac damage,” “staging,” and “classification” reporting on cardiac damage staging in an AS population and association with clinical outcomes. No language restrictions were applied. The flow chart demonstrating the study selection process (Preferred Reporting Items for Systematic Reviews and Meta-Analyses flow diagram) is reported in Supplemental Figure 1.

### Study selection and eligibility criteria

Two reviewers (O.M.A. and X.J.) independently and systematically reviewed the studies from the pooled search of databases, screened the abstracts, and confirmed their eligibility through full-text assessment. Disagreements were resolved by consensus with a third investigator (P.G.). Furthermore, we manually screened the references from articles of interest.

We included studies meeting the following inclusion criteria: 1) the population consisting of patients with AS, regardless of severity or symptom presentation; 2) patients included in these studies underwent AVR (surgical or transcatheter following baseline cardiac damage staging); 3) outcomes of interest included all-cause and/or CV death; 4) Kaplan-Meier curves were provided for each of the individual/grouped cardiac damage stages; and 5) the study design was retrospective/prospective cohorts, post-hoc randomized trial populations, monocentric/multicentric, with matched/unmatched populations. Animal studies, reviews, congress abstracts, editorials, letters, comments, case reports, and case series were excluded.

### Staging classification for cardiac damage

The presence and magnitude of extra-aortic valvular cardiac damage were evaluated based on baseline transthoracic echocardiography and, accordingly, patients were classified into 5 independent stages as proposed by Généreux et al^3^ prior to undergoing AVR:

• Stage 0—No signs of cardiac damage (ie, damage confined to aortic valve).
• Stage 1—LV damage (LV ejection fraction <50%, LV mass index >95 g/m^2^ for women or >115 g/m^2^ for men, or E/e′ >14).^35^^,^^36^
• Stage 2—Left atrial (LA) and/or mitral valve damage (LA volume index >34 mL/m^2^ or moderate mitral regurgitation grade ≥3 or presence of atrial fibrillation at the time of baseline echocardiography).^36^^,^^37^
• Stage 3—Tricuspid valve or pulmonary artery vasculature damage (systolic pulmonary artery pressure ≥60 mm Hg or tricuspid regurgitation grade ≥3).^37^
• Stage 4—Right ventricular damage (tricuspid annular plane systolic excursion <16 mm).^38^

If more than 1 of the included criteria were present, patients were hierarchically assigned to the highest (ie, worst) group accordingly.

### Endpoints of interest

The primary endpoint of this Kaplan-Meier-derived meta-analysis was all-cause death. Purposely, composite endpoints were not included in this meta-analysis as the definitions of heart failure hospitalization and readmissions varied across studies. Secondary endpoints were CV death and stroke. Populations studied were: 1) symptomatic severe AS; and 2) asymptomatic severe and asymptomatic moderate AS who underwent AVR.

### Data extraction

Individual patient data based on published Kaplan-Meier graphs from all included studies were reconstructed using the “curve approach.”^39^ We used the 2-stage approach described by Liu et al.^40^ In the first stage, raw data coordinates (time, survival probability) were extracted from each subgroup in each of the respective Kaplan-Meier curves.

Two investigators assessed the accuracy of the reconstructed patient data by comparing the survival probability in the published Kaplan-Meier curve at each read-in point with the corresponding reconstructed survival probability and estimated number of patients at risk.^40^

### Data pooling

The reconstructed individual patient data from the individual studies were merged to construct the final study datasets. Freedom from the respective outcomes was visually assessed using Kaplan-Meier estimates and analyzed using Cox proportional hazards models stratified by the cardiac damage stages (0, 1, 2, 3, and 4). Studies that provided merged staging categories (ie, stage 0-1 and stage 3-4) were reconstructed in a separate analysis.

### Risk for bias

The Risk of Bias in Nonrandomized Studies of Exposure tool was systematically used to assess included studies for risk of bias.^41^ The studies and their characteristics were classified into 5 groups: A (low risk for bias), B (moderate risk for bias), C (serious risk for bias), D (critical risk for bias), or E (no information/unclear). Two independent reviewers assessed the risk of bias. Disagreements were resolved by consensus among both reviewers.

### Statistical analysis

In the Kaplan-Meier-based meta-analysis, the mean ± SD survival times, median (IQR) survival times, and percentage of survival at different time points with 95% CIs were calculated. The differences in survival between the groups were assessed using the log-rank test for differences and a Cox proportional hazards regression model. Truncated survival analysis at 1 year was performed as a prespecified outcome, and the respective hazard ratios (HRs) and 95% CI were calculated. We also analyzed the data up to the longest follow-up. *P* values were 2-sided, and statistical significance was set at *P* <0.05. All analyses were completed with R version 4.2.1 (Foundation for Statistical Computing).

## Results

### Study population

From a total of 16 studies (N = 14,499) followed between 1998 and 2019,^4^^,^^6^, ^7^, ^8^, ^9^, ^10^^,^^13^, ^14^, ^15^, ^16^^,^^18^, ^19^, ^20^, ^21^, ^22^^,^^32^ 12 studies (n = 12,282) in symptomatic severe AS^4^^,^^6^^,^^7^^,^^9^^,^^10^^,^^14^^,^^15^^,^^18^, ^19^, ^20^, ^21^, ^22^ and 4 studies (n = 2,217) in asymptomatic severe/moderate AS^8^^,^^13^^,^^16^^,^^32^ undergoing AVR were included. Patients were categorized according to the cardiac damage staging (stages 0-4) based on echocardiographic data at baseline prior to AVR. Severe AS was defined according to current guidelines as a mean aortic valve gradient >40 mm Hg and/or aortic valve area <1.0 cm^2^ (or an indexed aortic valve area <0.6 cm^2^/m^2^) and/or a peak aortic jet velocity ≥4 m/s.^1^^,^^42^ Moderate AS was defined based on aortic valve area between 1.0 and 1.5 cm^2^.^43^

The overall clinical and echocardiographic characteristics of the included studies are summarized in Table 1 and Supplemental Table 1. The clinical and echocardiographic baseline characteristics of the pooled population according to merged and individual cardiac damage stages are presented in Supplemental Table 2 to 4. The descriptive data for the pooled population are presented in Table 2. The baseline characteristics of individual cardiac damage stages for symptomatic severe AS and asymptomatic severe/moderate AS are presented in Table 3 and Table 4, respectively. The overall internal validity of the analysis was considered low to moderate risk for bias, mostly because of the confounding caused by the differences in the unmatched populations (Supplemental Figure 2).

**Table 1:** Baseline Characteristics of Included Studies

First Author, Year (Study Name)	Center, Country, and Enrollment Period	N	Age, y	Male	BMI, kg/m^2^	DM	HLD	HTN	Previous CABG	MI	NYHA Functional Class ≥III	Lung Disease^a^	STS-PROM Score	AF/AFL
Fukui et al^6^, 2019	Single, USA, 2011-2017	689	82.4 ± 7.6	351 (50.9)	28.2 ± 6.4	283 (41.1)	541 (78.5)	617 (89.6)	199 (28.9)	263 (38.2)	543 (78.8)	264 (38.3)	8.2 ± 4.7	321 (46.6)
Vollema et al^7^, 2019	Multicenter, Leiden and Singapore, 1999-2017	1,189	73.4 ± 10.8	624 (53)	25.5 ± 4.6	317 (27)	790 (66)	857 (72)	—	189 (16)	393 (33)	129 (11)	—	354 (30)
Berkovitch et al^9^, 2020	Multicenter, Israel, 2008-2018	2,608	82 ± 7	1,186 (45)	—	964 (40)	1,990 (76.3)	2,234 (85.7)	—	—	2,079 (79.7)	414 (15.9)	5.3 ± 3.8	—
Maeder et al^10^, 2020	Single, Switzerland, 2011-2016	421	75 ± 10	248 (59)	—	91 (21.6)	—	—	—	—	138 (32.8)	53 (12.6)	—	47 (11.2)
Avvedimento et al^18^, 2021 (EffecTAVI Registry)	Single, Italy, 2014-2019	262	79.5 ± 6	100 (38.2)	—	87 (33.2)	159 (60.7)	229 (87.4)	—	47 (17.9)	151 (57.6)	71 (27.1)	5 ± 3.1	—
Okuno et al^15^, 2021	Single, Switzerland, 2007-2016	1,133	82.1 ± 6.3	557 (49.2)	—	—	—	946 (83.5%)	203 (17.9)	—	775 (68.5)	162 (14.3)	6.07 ± 4.2	—
Schewel et al^14^, 2021	Single, Germany, 2008-2017	1,400	81.5 ± 6.8	648 (46.3)	—	424 (30.3)	522 (37.3)	1,168 (83.4)	168 (12)	—	1,225 (87.5)	231 (16.5)	6.6± (IQR: 5.9-7.1)	653 (46.6)
Hirasawa et al^20^, 2021	Single, the Netherlands, 2007-2019	405	80 ± 7	212 (52)	—	115 (28)	264 (65)	309 (76)	—	—	235 (58)	90 (22)	—	—
Généreux et al^4^, 2022 (PARTNER II & III Trials)	Multicenter	1,974	80.7 ± 7.76	1,086 (55)	—	626 (31.7)	—	1,804 (91.5)	349 (17.7)	284 (14.4)	1,299 (65.8)	434 (22.1)	5.8 ± 4.2	—
Shamekhi et al^21^, 2022	Multicenter, Germany, 2008-2019	933	80.5 ± 6.3	470 (50.3)	—	271 (29.0)	—	809 (86.7)	144 (15.4)	—	789 (84.6)	205 (22)	5.2 ± 4.3	387 (41.5)
Pellegrini et al^22^, 2022	Single, Germany, 2011-2016	841	80.7 ± 5.5	443 (52.7)	—	240 (28.5)	—	764 (90.8)	83 (9.9)	—	568 (67.5)	133 (15.8)	—	—
Zhu et al^19^, 2022 (TORCH Registry)	Single, (China), 2013-2019	464	76.1 ± 6.5	249 (58.3)	22.7 ± 3.5	97 (22.7)	93 (21.8)	233 (54.6)	2 (0.5)	—	377 (88.3)	99 (23.2)	7.18 ± 5.61	72 (16.9)
Tastet et al^8^, 2019^b^	Multicenter, North America, France and Netherlands, 1998-2017	735	71 ± 14	442 (60.1)	27 ± 5	181 (24.8)	—	516 (70.4)	62 (8.6)	77 (10.6)	0	96 (13.1)	—	152 (20.8)
Amanullah et al^16^, 2021^b^	Multicenter, Leiden, and Singapore, 2001-2018	1,245	70.9 ± 12.3	622 (50)	25.5 ± 5.9	435 (34.9)	998 (80.2)	986 (79.2)	—	215 (17.3)	145 (11.6)	70 (5.6)	—	298 (23.9)
Park et al^13^, 2021^b^ (RECOVERY Trial)	Multicenter, Korea, 2010-2015	145	—	71 (48.9)	—	20 (13.8)	83 (57.2)	79 (54.5)	—	—	—	—	—	9 (6.2)
Sevilla et al^32^, 2023^b^	Single, Spain	96	71 ± 11	69 (72)	—	20 (21)	53 (55)	68 (71)	—	10 (10)	—	—	—	—

Values are as n (%) or mean ± SD.

AF = atrial fibrillation; AFL = atrial flutter; BMI = body mass index; CABG = coronary artery bypass graft; HLD = dyslipidemia/hyperlipidemia; MI = myocardial infarction; STS-PROM = Society of Thoracic Surgeons Predicted Risk of Mortality; TAVR = transcatheter aortic valve replacement.

a Included patients with chronic obstructive pulmonary disease, chronic bronchitis, or emphysema.

b Population was moderate or severe asymptomatic aortic stenosis.

**Table 2:** Pooled Estimates of Characteristics in Symptomatic Severe and Asymptomatic Moderate/Severe Patients From All Included Studies

Variable	Symptomatic Severe AS (N = 12,282)		Asymptomatic Moderate/Severe AS (N = 2,217)	
	Proportion/Mean (95% CI)	I^2^, *P* Value	Proportion/Mean (95% CI)	I^2^, *P* Value
Age, y	79.5 (77.8-81.2)	99.0%, <0.001	70.9 (70.4-71.5)	0%, 0.872
Male, %	50.5% (47.9-53.1)	87.5%, <0.001	57.3% (48.9-65.6)	91.7%, <0.001
DM, %	29.9% (26.6-33.1)	92.7%, <0.001	23.9% (14.8-32.9)	94.9%, <0.001
HLD, %	57.8% (41.6-74)	99.5%, <0.001	64.6% (45.8-83.4)	95.9%, <0.001
HTN, %	81.8% (77.3-86.2)	98.0%, <0.001	69.3% (60.6-78)	93.6%, <0.001
Previous cardiac surgery, %	14.5% (6.9-22.2)	99.4%, <0.001	8.4% (6.4-10.4)	—
MI, %	21.5% (12.7-30.3)	97.9%, <0.001	13% (7.6-18.3)	90.2%, <0.001
NYHA functional class ≥III, %	66.3% (56.7-75.9)	99.4%, <0.001	11.6% (9.9-13.4)	—
Lung disease, %	19.7% (16.3-23)	95.8%, <0.001	9.3% (2-16.6)	96.4%, <0.001
Pre-TAVR AF/AFL, %	31.9% (19.6-44.1)	99%, <0.001	17.1% (7.7-26.4)	96.6%, <0.001
Past CVA/TIA, %	10.4% (8.7-12.1)	79.9%, <0.001	1.4% (0.7-2)	—
CAD, %	58.7% (55.3-62.1)	89.3%, <0.001	34.2% (7-61.4)	99.5%, <0.001
CKD, %	51.2% (45.2-57.2)	95.4%, <0.001	20% (17.1-22.9)	—
BMI, kg/m^2^	26.1 (25.4-26.7)	95.0%, <0.001	26.2 (24.8-27.7)	97.2%, <0.001
STS-PROM score, %	6.1 (5.6-6.7)	98.6%, <0.001	—	—
LVMI, g/m^2^	126.7 (113.5-140)	99.4%, <0.001	114.9 (105.5-124.3)	98.8%, <0.001
LVEF, %	52.4 (49.3-55.5)	96.9%, <0.001	60.1 (56.5-63.7)	97.3%, <0.001
PASP, mmHg	43.7 (38.3-49.1)	99.1%, <0.001	37.8 (32.2-43.3)	97.9%, <0.001
AV gradient, mmHg	42.9 (41-44.7)	94.7%, <0.001	28 (24.2-31.7)	97.5%, <0.001
AVA, cm^2^	0.7 (0.7-0.8)	96.6%, <0.001	1.1 (1-1.2)	98.9%, <0.001
LAVI, mL/m^2^	48.5 (41.2-55.8)	99.6%, <0.001	41.6 (32.5-50.6)	99.4%, <0.001

AF = atrial fibrillation; AFL = atrial flutter; AV = aortic valve; AVA = aortic valve area; BMI = body mass index; CAD = coronary artery disease; CKD = chronic kidney disease; CVA/TIA = cerebrovascular accident/transient ischemic attack; DM = diabetes mellitus; HLD = dyslipidemia/hyperlipidemia; HTN = hypertension; LAVI = left atrial volume index; LVEF = left ventricular ejection fraction; LVMI = left ventricular mass index; MI = myocardial infarction; PASP = pulmonary artery systolic pressure; STS-PROM = Society of Thoracic Surgeons Predicted Risk of Mortality.

**Table 3:** Pooled Estimates of Characteristics in Symptomatic Severe AS, According to Cardiac Damage Stage

	Stage 0 (n = 1,391)		Stage 1 (n = 1,988)		Stage 2 (n = 4,231)		Stage 3 (n = 1,688)		Stage 4 (n = 1,168)	
	Proportion/Mean (95% CI)	I^2^, *P* Value	Proportion/Mean (95% CI)	I^2^, *P* Value	Proportion/Mean (95% CI)	I^2^, *P* Value	Proportion/Mean (95% CI)	I^2^, *P* Value	Proportion/Mean (95% CI)	I^2^, *P* Value
Age, y	78.4 (76.2-80.6)	97.1%, <0.001	78.8 (76.6-80.9)	97.1%, <0.001	80.1 (78.2-82)	97.7%, <0.001	80.7 (78.3-83)	95.1%, <0.001	78.8 (77.4-80.3)	79.9%, <0.001
Male, %	51.1% (43.4-58.8)	82.5%, <0.001	51.7% (46.5-56.9)	83.1%, <0.001	50.8% (47.8-53.9)	74.4%, <0.001	47.7% (42.6-52.8)	74%, <0.001	59.2% (53.4-65.1)	71.3%, <0.001
DM, %	29.9% (25.2-34.7)	60%, 0.014	31.2% (26.8-35.5)	78.9%, <0.001	30.4% (26.9-33.9)	83.5%, <0.001	29.3% (24.5-34.1)	75.5%, <0.001	35.8% (30.8-40.9)	50.2%, 0.034
HLD, %	54.9% (29.3-80.4)	98.6%, <0.001	58.5% (40.5-76.6)	98.2%, <0.001	58.3% (43.6-73)	98.4%, <0.001	56.7% (40.4-73.0)	96.6%, <0.001	54.0% (37.1-70.8)	94.2%, <0.001
HTN, %	80.8% (73.6-87.9)	88.1%, <0.001	82.1% (77.6-86.6)	88.6%, <0.001	84.2% (80.1-88.3)	94%, <0.001	81.5% (75.1-87.8)	93.4%, <0.001	76.3% (69.8-82.8)	84.8%, <0.001
Previous cardiac surgery, %	6.6% (2.0-11.2)	57%, 0.072	9.9% (5.6-14.2)	85.1%, <0.001	17.9% (11.3-24.5)	95.9%, <0.001	15.7% (9.1-22.4)	89.1%, <0.001	25.0% (11.6-38.3)	96.2%, <0.001
MI, %	9% (3.4-14.5)	50.9%, 0.153	17.5% (8.8-26.2)	89.3%, <0.001	23.2% (10.9-35.5)	97.9%, <0.001	20.1% (7.8-32.5)	91.5%, <0.001	30.2% (21.7-38.7)	68.2%, 0.024
NYHA functional class ≥III, %	61.4% (48.2-74.7)	96%, <0.001	67.4% (53.5-81.3)	98.5%, <0.001	72.0% (59.5-84.5)	99.1%, <0.001	66.9% (41.9-91.9)	99.4%, <0.001	75.9% (67.4-84.4)	92.4%, <0.001
Lung disease, %	16.8% (13.7-19.8)	39.1%, 0.131	18.5% (13.7-23.3)	89.2%, <0.001	20.3% (14.9-25.7)	95.5%, <0.001	23% (19.6-26.3)	57.1%, 0.022	21.1% (16.1-26.1)	74%, <0.001
Pre-TAVR AF/AFL, %	19.5% (0-42.0)	95.6%, <0.001	27.5% (11.8-43.2)	97.4%, <0.001	42.0% (29.8-54.2)	97.1%, <0.001	52.6% (44.2-61.0)	77.3%, 0.001	50.5% (36.6-64.4)	87.6%, <0.001
Past CVA/TIA, %	8.6% (4.8-12.4)	69.6%, 0.006	10.1% (5.8-14.4)	76%, 0.006	12.3% (8.2-16.5)	87.8%, <0.001	13.4% (8.4-18.3)	78.5%, 0.003	12.1% (9.7-14.4)	1.4%, 0.413
CAD, %	53.1% (43.4-62.8)	73.9%, 0.001	59.1% (48.6-69.6)	94.3%, <0.001	60.4% (52.4-68.4)	95.4%, <0.001	58.9% (50.5-67.2)	85.9%, <0.001	64.7% (58.1-71.3)	78.1%, <0.001
CKD, %	37.6% (24.1-51.2)	82.5%, 0.003	48.5% (40.3-56.7)	86.5%, 0.001	54.5% (45.4-63.5)	93.4%, <0.001	60.2% (48.3-72.0)	91.2%, 0.001	58.0% (39.9-76.1)	96%, <0.001
BMI, kg/m^2^	25.0 (23.4-26.6)	95.6%, <0.001	25.7 (23.6-27.7)	95.0%, <0.001	26.2 (24.6-27.8)	96.2%, <0.001	25.2 (23.1-27.2)	96.1%, 0.001	25.9 (23.9-27.9)	94.6%, <0.001
STS-PROM score, %	4.3 (3.4-5.2)	97.1, <0.001	5.4 (4.4-6.5)	97.1%, <0.001	6.7 (5.7-7.6)	96.3%, <0.001	8.0 (7.1-8.9)	90.8%, 0.001	7.4 (6.3-8.5)	74.3%, 0.004
LVMI, g/m^2^	98.9 (52.8-145)	99.4%, <0.001	123.5 (100.4-146.7)	99.2%, <0.001	134.5 (106-163)	99.6%, <0.001	134.9 (106.1-163.6)	99.1%, <0.001	134.9 (107.4-162.4)	98.0%, <0.001
LVEF, %	60.4 (56.3-64.5)	95.0%, <0.001	57.0 (55.8-58.3)	56.3%, 0.077	55 (53.6-56.4)	78.2%, 0.003	50.1 (47.5-52.7)	76.3%, <0.001	39.4 (34.4-44.5)	84.9%, <0.001
PASP, mm Hg	31.1 (22.9-39.2)	98.0%, <0.001	36.1 (28.8-43.4)	97.4%, <0.001	38.4 (34.1-42.8)	98.6%, <0.001	59 (54.5-63.4)	91.6%, <0.001	52.9 (41.9-63.9)	97.9%, <0.001
AV gradient, mm Hg	42.4 (39.3-45.5)	86.8%, <0.001	45.3 (41.5-49.2)	92.6%, <0.001	45.8 (42.6-48.9)	95.9%, <0.001	42.3 (37.8-46.8)	95.3%, <0.001	36.5 (33.9-39.2)	78.6%, 0.003
AVA, cm^2^	0.8 (0.8-0.8)	45.7%, 0.158	0.7 (0.7-0.8)	95.1%, <0.001	0.7 (0.7-0.8)	98.1%, <0.001	0.7 (0.6-0.8)	97.2%, <0.001	0.7 (0.6-0.7)	88.4%, <0.001
LAVI, mL/m^2^	34.9 (15.1-54.7)	99.6%, <0.001	33.5 (20.3-46.7)	99.7%, <0.001	55.1 (45.4-64.8)	99.0%, <0.001	63.1 (50.9-75.3)	94.5%, <0.001	60.6 (50.2-71.1)	91.2%, <0.001

AF = atrial fibrillation; AFL = atrial flutter; AS = aortic stenosis; AV = aortic valve; AVA = aortic valve area; BMI = body mass index; CAD = coronary artery disease; CKD = chronic kidney disease; CVA/TIA = cerebrovascular accident/transient ischemic attack; DM = diabetes mellitus; HLD = dyslipidemia/hyperlipidemia; HTN = hypertension; LAVI = left atrial volume index; LVEF = left ventricular ejection fraction; LVMI = left ventricular mass index; MI = myocardial infarction; PASP = pulmonary artery systolic pressure; STS-PROM = Society of Thoracic Surgeons Predicted Risk of Mortality.

**Table 4:** Pooled Estimates of Characteristics in Asymptomatic Moderate/Severe AS, According to Cardiac Damage Stage

	Stage 0 (n = 272)^a^		Stage 1 (n = 529)^a^		Stage 2 (n = 898)^a^		Stage 3 (n = 148)^a^		Stage 4 (n = 133)^a^	
	Proportion/Mean (95% CI)	I^2^, *P* Value	Proportion/Mean (95% CI)	I^2^, *P* Value	Proportion/Mean (95% CI)	I^2^, *P* Value	Proportion/Mean (95% CI)	I^2^, *P* Value	Proportion/Mean (95% CI)	I^2^, *P* Value
Age, y	65.1 (63.1-67)	6.6%, 0.301	69.3 (64.9-73.7)	93%, <0.001	72.8 (70.6-75.1)	88%, 0.004	72.9 (67.3-78.5)	59.6%, 0.116	73.4 (71.4-75.4)	0%, 0.370
Male, %	64.3% (58.7-70.0)	—	53.5% (46.6-60.5)	60.7%, 0.111	53.8% (39.6-68.1)	94.7%, <0.001	43.9% (35.9-51.9)	0%, 0.988	54.9% (43.6-66.3)	42.1%, 0.189
DM, %	21.4% (15.9-26.8)	19.9%, 0.264	28.7% (13.8-43.6)	93.3%, <0.001	32.5% (26.5-38.5)	73.5%, 0.052	25.5% (2.2-48.8)	84.9%, 0.010	33.5% (9.9-57.0)	88.9%, 0.003
HLD, %	77.3% (70.9-83.7)	—	82.6% (78.6-86.7)	—	77.9% (74.4-81.5)	—	78.8% (71.8-85.8)	—	91.9% (86.1-97.6)	—
HTN, %	63.8% (51-76.6)	79.2%, 0.028	72% (56-88)	93.8%, <0.001	79.2% (75.1-83.4)	56.9%, 0.128	76.5% (69.6-83.3)	0%, 0.599	80.8% (66.3-95.2)	74.9%, 0.046
Previous cardiac surgery, %	2.8% (0-5.8)	—	6.2% (2.8-9.5)	—	10.6% (7.5-13.7)	—	12.5% (0-28.7)	—	12.8% (3.2-22.3)	—
MI, %	6.7% (1.1-12.3)	67.7%, 0.078	14% (4.6-23.5)	90.4%, 0.001	15.1% (7.3-22.8)	91.1%, 0.001	12.5% (3.4-21.5)	49.9%, 0.158	19.8% (6.1-33.5)	76.2%, 0.04
NYHA functional class ≥III, %	1.3% (0-4.3)	76.2%, 0.040	3.6% (0-10.9)	96.1%, <0.001	13.2% (10.3-16.1)	—	10% (0-29.3)	92.6%, <0.001	10.6% (-11-32.3)	95.5%, <0.001
Lung disease, %	4.5% (2-6.9)	—	8.9% (6.5-11.4)	0.8%, 0.315	10.4% (0.8-20.1)	95.3%, <0.001	4% (0.8-7.1)	0%, 0.695	10.9% (−7.6-29.4)	89.3%, 0.002
Pre-TAVR AF/AFL, %	0.7% (0-2.5)	55.1%, 0.135	2.4% (0-7.4)	93.9%, <0.001	31.5% (28.5-34.5)	0%, 0.557	54.7% (46.7-62.8)	0%, 0.688	49.6% (41.1-58.1)	0%, 0.806
Past CVA/TIA, %	4.6% (0.7-8.5)	—	10.3% (6-14.5)	—	12.2% (8.9-15.6)	—	25% (3.8-46.2)	—	10.6% (1.8-19.5)	—
CAD, %	26.3% (7-45.6)	93.1%, <0.001	34% (1.5-66.5)	98.6%, <0.001	36.1% (9.0-63.3)	98.8%, <0.001	36.1% (21.7-50.4)	46.5%, 0.172	41.5% (6.3-76.7)	94.9%, <0.001
CKD, %	13.8% (7.3-20.2)	-	11.8% (7.3-16.3)	—	24.7% (20.3-29.1)	—	18.8% (0-37.9)	—	31.9% (18.6-45.2)	—
BMI, kg/m^2^	26.9 (26.3-27.6)	0%, 0.739	26.9 (26.3-27.6)	0%, 0.739	26.9 (24.9-29)	97.4%, <0.001	24.5 (22.2-26.8)	69.6%, 0.070	24.7 (22.2-27.1)	88.5%, 0.003
STS-PROM score, %	—	—	—	—	—	—	—	—	—	—
LVMI, g/m^2^	89.1 (87.4-90.8)	0%, 0.371	89.1 (87.4-90.8)	0%, 0.371	125.6 (123.2-128)	0%, 0.723	119.9 (102.7-137.2)	78.7%, 0.030	123.7 (117.4-130)	0%, 0.547
LVEF, %	65.5 (64.4-66.6)	67.1%, 0.081	65.5 (64.4-66.6)	67.1%, 0.081	60.6 (55.8-65.4)	98.3%, <0.001	59 .0 (49.4-68.6)	94.2%, <0.001	54.1 (38.5-69.7)	98.3%, <0.001
PASP, mm Hg	30.1 (28.3-31.9)	81.1%, 0.022	30.1 (28.3-31.9)	81.1%, 0.022	34.5 (33.5-35.5)	66.3%, 0.085	52.7 (50.3-55.2)	0%, 0.740	41.5 (34.6-48.5)	85.7%, 0.008
AV gradient, mm Hg	27.8 (23.9-31.7)	86.9%, 0.006	27.8 (23.9-31.7)	86.9%, 0.006	30.2 (19-41.5)	99.4%, <0.001	24.3 (20-28.6)	49.4%, 0.160	26.3 (9.5-43)	97.7%, <0.001
AVA, cm^2^	1.1 (0.9-1.4)	98.9%, <0.001	1.1 (0.9-1.4)	98.9%, <0.001	1.1 (0.8-1.4)	99.8%, <0.001	1.0 (0.8-1.3)	95.6%, <0.001	1 (0.7-1.3)	98.5%, <0.001
LAVI, mL/m^2^	25.9 (25.3-26.6)	0%, 0.875	25.9 (25.3-26.6)	0%, 0.875	45.5 (44.5-46.4)	0%, 0.345	64.7 (56.7-72.7)	11.8%, 0.116	51 (45.1-56.8)	68.4%, 0.075

AF = atrial fibrillation; AFL = atrial flutter; AS = aortic stenosis; AV = aortic valve; AVA = aortic valve area; BMI = body mass index; CAD = coronary artery disease; CKD = chronic kidney disease; CVA/TIA = cerebrovascular accident/transient ischemic attack; DM = diabetes mellitus; HLD = dyslipidemia/hyperlipidemia; HTN = hypertension; LAVI = left atrial volume index; LVEF = left ventricular ejection fraction; LVMI = left ventricular mass index; MI = myocardial infarction; NYHA = New York Heart Association; PASP = pulmonary artery systolic pressure; STS-PROM = Society of Thoracic Surgeons Predicted Risk of Mortality.

a Staged baseline characteristics/echocardiographic parameters were only available in 2 included studies.

### Symptomatic severe AS and mortality

**Figure 1:**
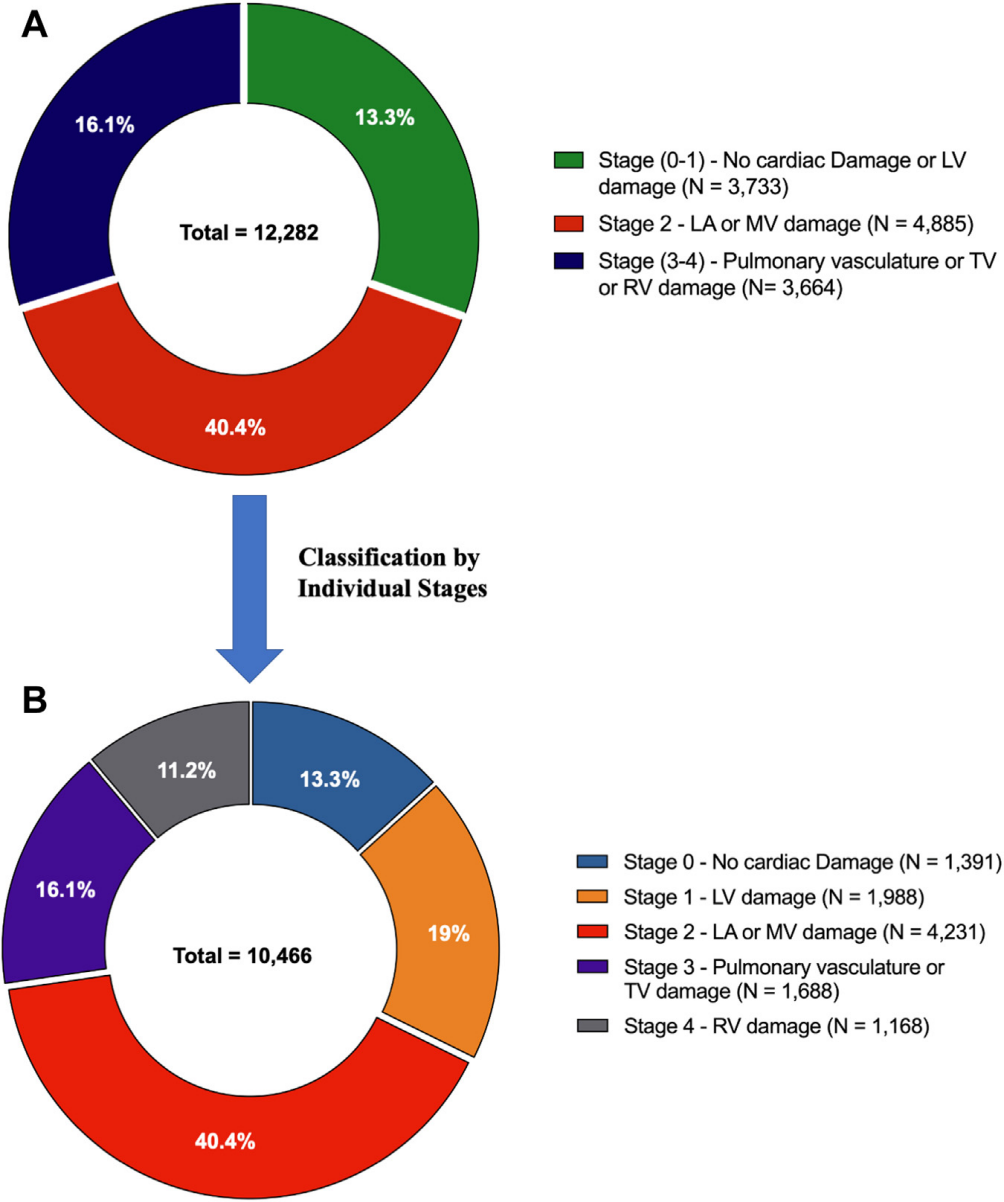
**Distribution of Cardiac Damage Stages in Severe Symptomatic Aortic Stenosis** (A) distribution of grouped cardiac damage stages (Stage 0-1, Stage 2, and Stage 3-4), (B) distribution of individual cardiac damage stages (Stages 0, 1, 2, 3, and 4). LA = left atrial; LV = left ventricular; MV = mitral valve; RV = right ventricular; TV = tricuspid valve.

Among the 12 included studies (11 observation studies and 1 publication with 2 randomized controlled trials pooled—PARTNER II and III [Placement of AoRTic TraNscathetER Valves in Low, Intermediate and High risk patients])^4^^,^^6^^,^^7^^,^^9^^,^^10^^,^^14^^,^^15^^,^^18^, ^19^, ^20^, ^21^, ^22^ (N = 12,282), 9 studies presented data on individual cardiac damage stages (Stage 0-4), while 3 studies merged cardiac damage stages (ie, stage 0-1 and stage 3-4) owing to small sample sizes in individual stages. In symptomatic severe AS population, the mean age was 79.5 years (95% CI: 77.8-81.2 years), 50.5% were male, and 66.3% were in NYHA functional class III-IV. The pooled prevalence of the grouped and individual original cardiac damage stages are presented in Figure 1. According to the staging classification, 1,391 (13.3%) patients were in stage 0, 1,988 (19%) patients were in stage 1, 4,231 (40.4%) patients were in stage 2, 1,688 (16.1%) patients were in stage 3, and 1,168 (11.2%) patients were in stage 4. Patients in the more advanced stages of cardiac damage were older, presented with an increasing proportion of NYHA functional class III-IV, and had a higher prevalence of comorbidities, including coronary artery disease (CAD), chronic kidney disease, atrial fibrillation, previous cardiac surgery, myocardial infarction, and prior stroke. The clinical and echocardiographic baseline characteristics of the pooled population according to merged and individual cardiac damage stages are presented in Tables 2 and 3 and Supplemental Tables 2 to 4. During a median follow-up of 1 year (IQR: 0.6-2.0 years), there were 2,124 overall deaths reported resulting in a pooled crude rate of death of 35.6% (95% CI: 33.8%-37.3%).

**Figure 2:**
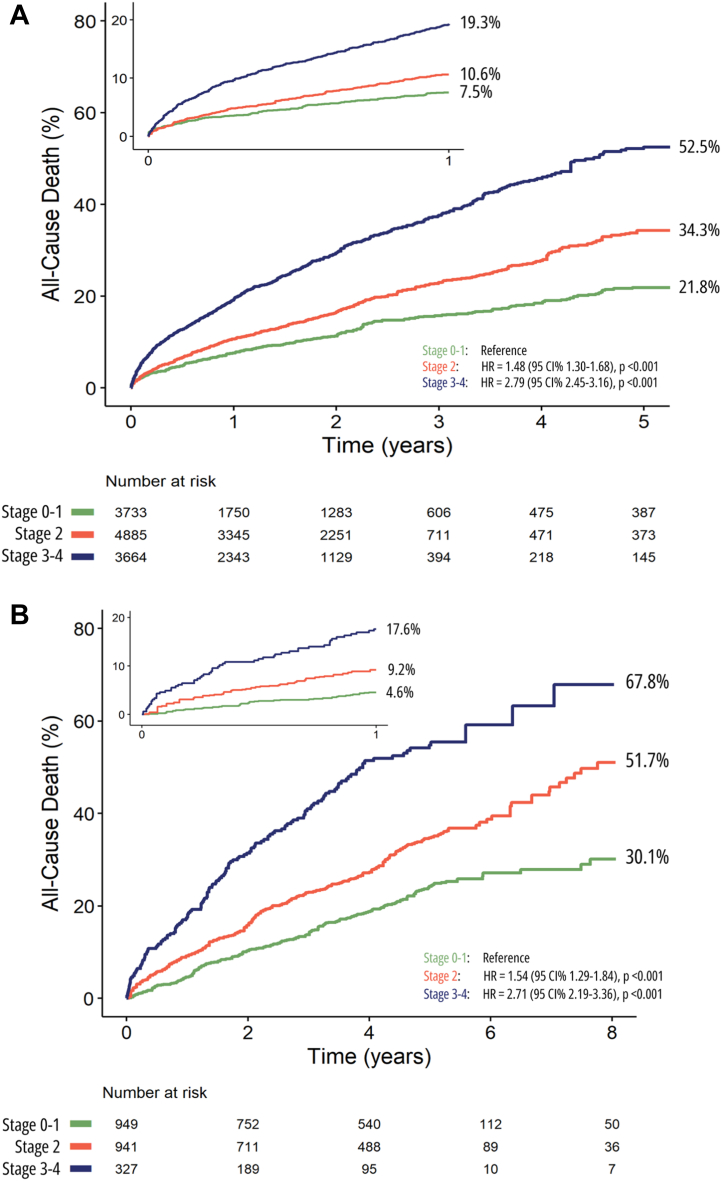
Reconstructed Kaplan-Meier Analyses According to Cardiac Damage for Severe Symptomatic Aortic Stenosis Following AVR (A) grouped cardiac damage stages (Stage 0-1, Stage 2, and Stage 3-4), (B) individual cardiac damage stages (Stages 0, 1, 2, 3, and 4). AVR = aortic valve replacement.

Based on individual cardiac damage stages (stages 0, 1, 2, 3, and 4) following the Généreux et al^3^ staging classification, a total of 10,466 patients (stage 0: 1,391 patients; stage 1: 1,988 patients; stage 2: 4,231 patients; stage 3: 1,688 patients; and stage 4: 1,168 patients) from 9 studies were pooled (Figure 1). Mortality at 1-year increased in patients with higher cardiac damage at baseline. In patients who had cardiac damage classified as stage 0, 1-year mortality was 6.6%. Whereas patients classified as stage 1, 2, 3, and 4 had a 1-year mortality of 8.5%, 11.0%, 17.7%, and 19.6%, respectively (1-year HR in stage 1: 1.28 [95% CI: 0.96-1.71], *P* = 0.088; stage 2: 1.71 [95% CI: 1.32-2.21], *P* < 0.001; stage 3: 2.79 [95% CI: 2.13-3.64], *P* < 0.001; and stage 4: 3.56 [95% CI: 2.79-4.71]; *P* < 0.001) (Central Illustration). At the final follow-up (5 years), all-cause mortality was 24.0%, 27.7%, 38.0%, 56.3%, and 57.3% in patients with cardiac damage stage 0, 1, 2, 3, and 4, respectively (HR in stage 1: 1.30 [95% CI: 1.03-1.64], *P* = 0.029; stage 2: 1.74 [95% CI: 1.41-2.16], *P* < 0.001; stage 3: 2.92 [95% CI: 2.35-3.64], *P* < 0.001; and stage 4: 3.51 [95% CI: 2.79-4.41], *P* < 0.001) (Figure 2).

**Central Illustration:**
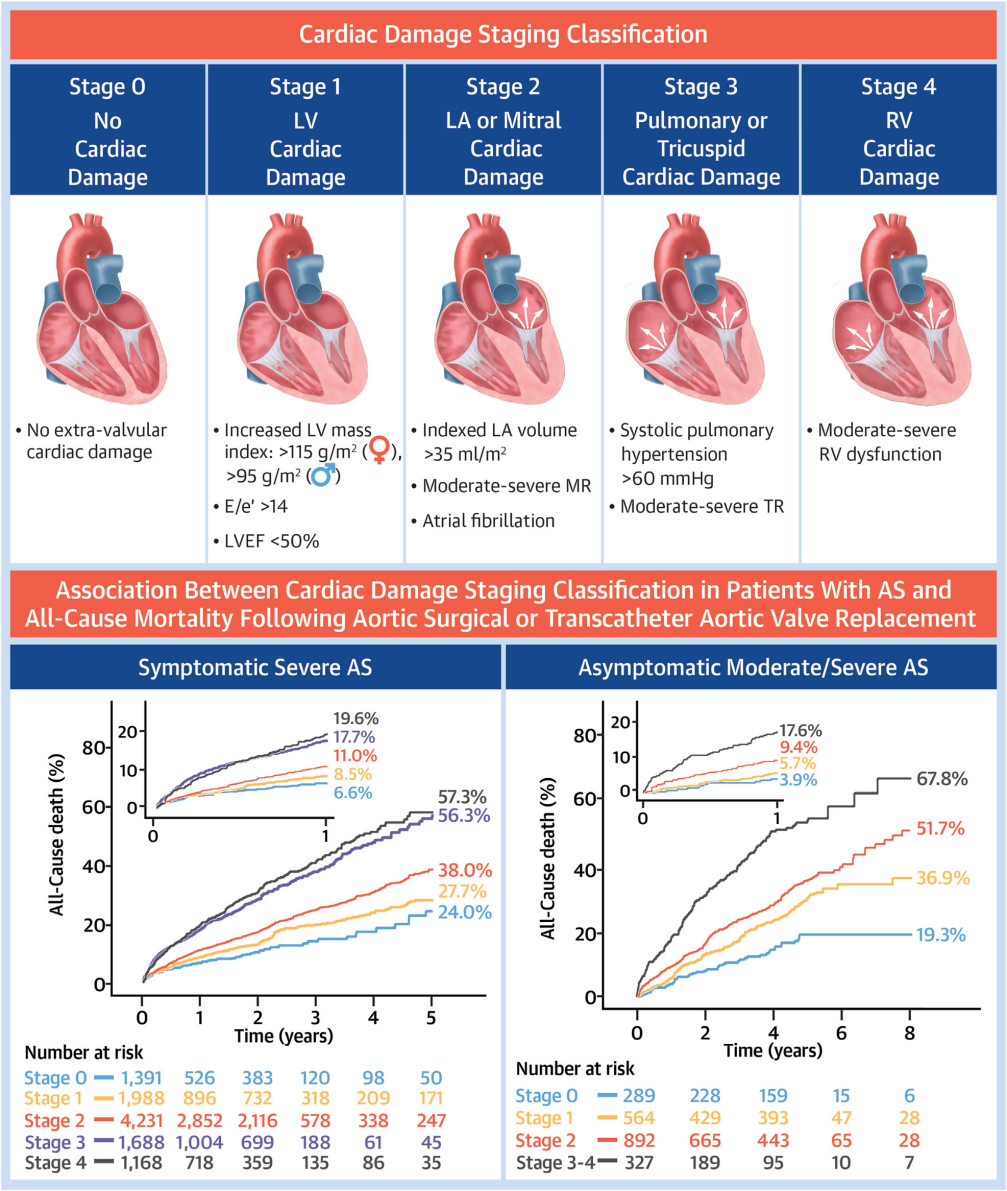
Cardiac Damage Staging Across Symptomatic and Severity Spectrum of Aortic Stenosis Undergoing AVR AS = aortic stenosis; AVR = aortic valve replacement; LA = left atrial; LV = left ventricular; LVEF = left ventricular ejection fraction; MR = mitral regurgitation; RV = right ventricle; TR = tricuspid regurgitation.

Based on merged stages (stages 0-1, 2, and 3-4) following the Généreux et al^3^ staging classification, 12,282 patients (stage 0-1: n = 3,733; stage 2: n = 4,885; and stage 3-4: n = 3,664) from 12 studies were pooled (Figure 1, Supplemental Appendix).

### Asymptomatic moderate and severe AS and mortality

**Figure 3:**
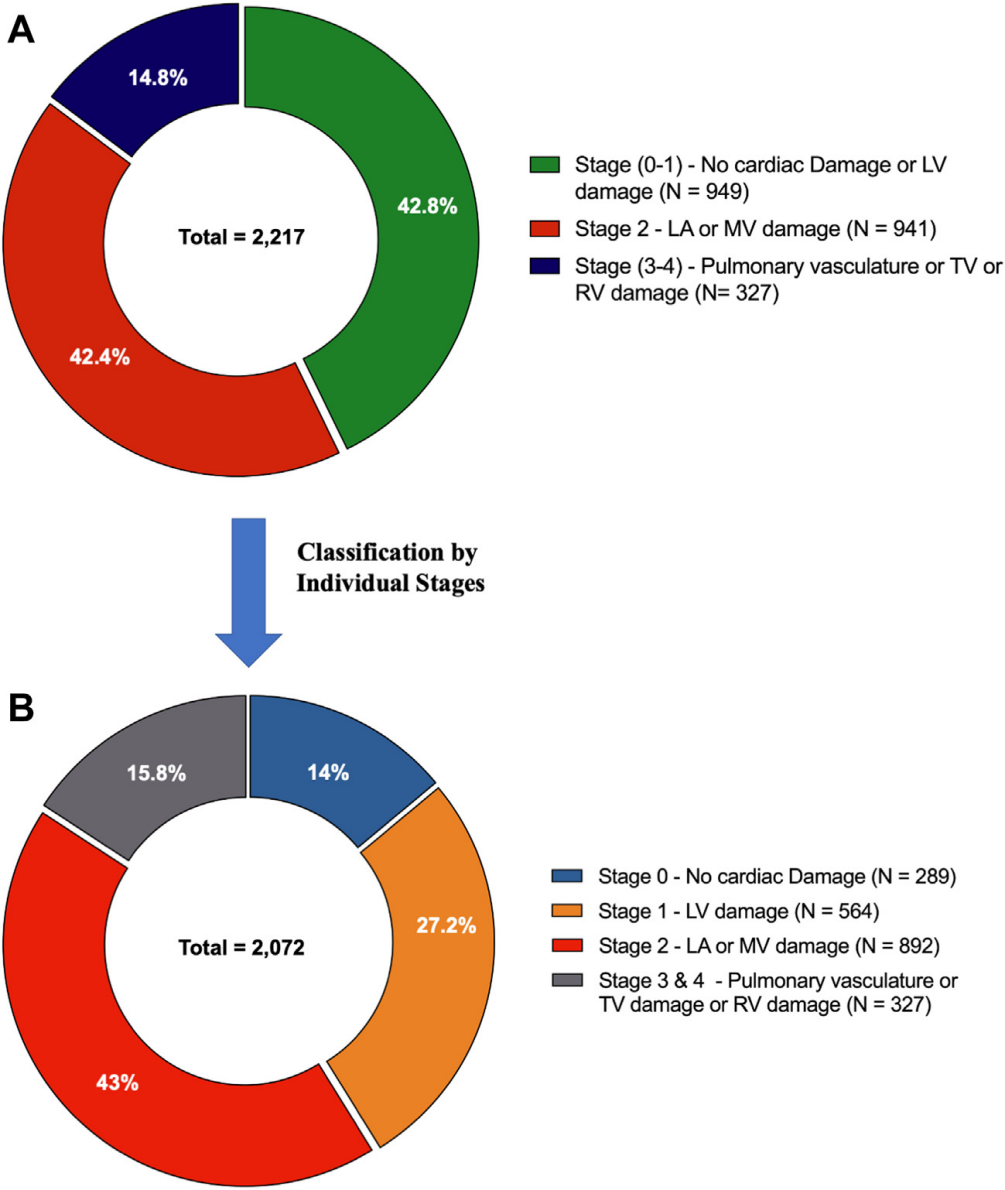
Distribution of Cardiac Damage Stages in Asymptomatic Moderate/Severe Aortic Stenosis (A) grouped cardiac damage stages (Stage 0-1, Stage 2, and Stage 3-4), (B) individual cardiac damage stages (Stages 0, 1, 2, 3, and 4). LA = left atrial; LV = left ventricular.

In total, 4 studies (3 observational and 1 randomized controlled trial—RECOVERY [Early Surgery Versus Conventional Treatment in Very Severe Aortic Stenosis] trial) (N = 2,217) reported on outcomes in relation to cardiac damage staging in asymptomatic moderate and asymptomatic severe AS undergoing AVR.^8^^,^^13^^,^^16^^,^^32^ In asymptomatic moderate/severe AS, the mean age of the population was 70.9 (IQR: 70.4-71.5) years, and 57.3% were male; an expectedly low proportion (11.6%) presented with NYHA functional class III-IV symptoms. According to the staging classification, 289 (14%) patients were in stage 0, 564 (27.2%) patients were in stage 1, 892 (43%) patients were in stage 2, and 327 (15.8%) patients were in stage 3 and stage 4 (Figure 3). Asymptomatic moderate/severe AS patients in advanced stages were older, with a greater proportion presenting with NYHA functional class III-IV and a higher prevalence of CAD, chronic kidney disease, prior cardiac surgery, myocardial infarction history, and a lower LV ejection fraction (Table 4). During a median follow-up of 4.1 years (IQR: 1.9-5.2 years), 617 deaths were reported, with a pooled crude rate of death of 33.0% (95% CI: 30.8%-35.2%).

**Figure 4:**
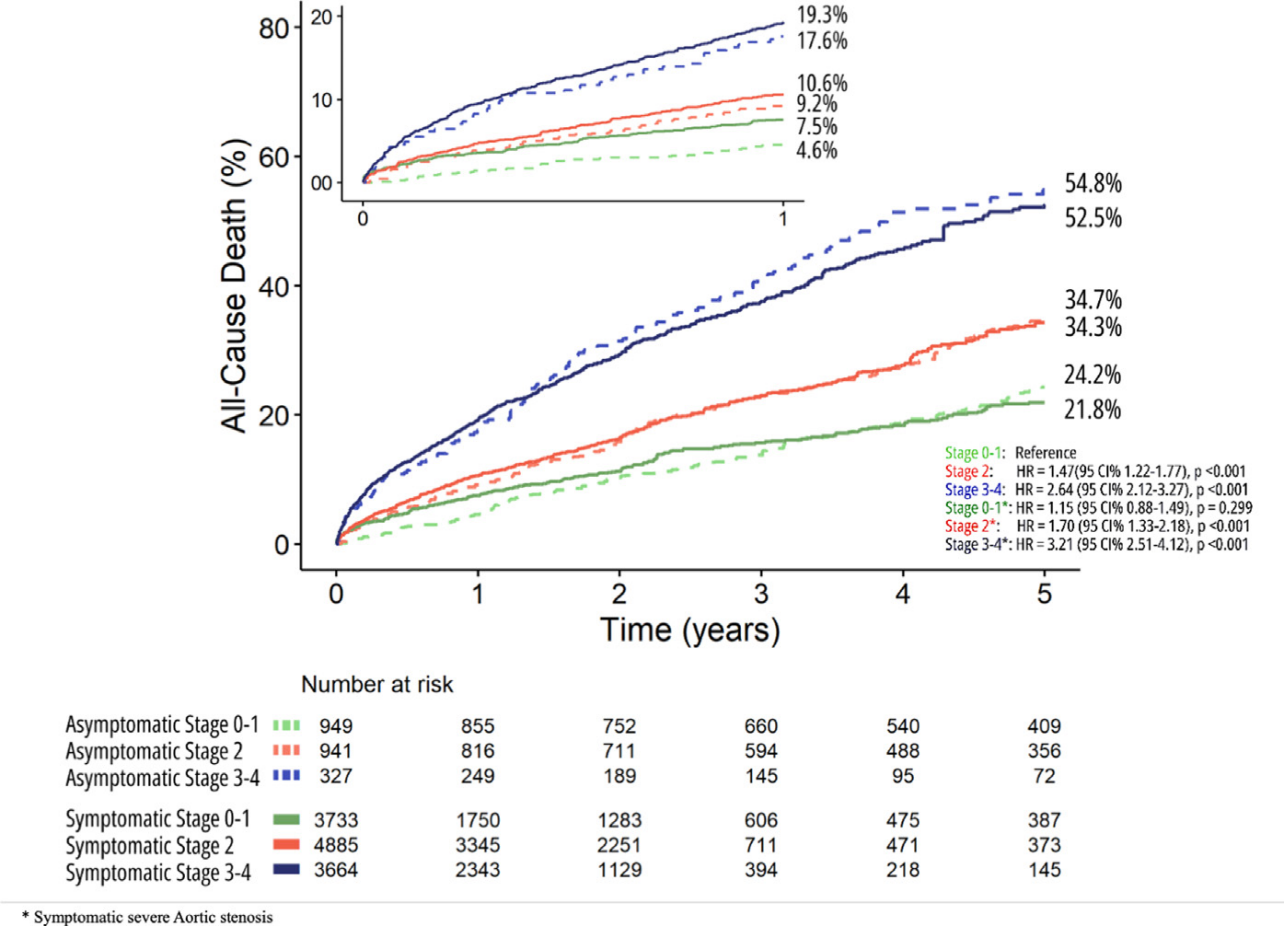
Reconstructed Kaplan-Meier Analyses According to Cardiac Damage for All-Cause Mortality in Asymptomatic Moderate/Severe Aortic Stenosis Undergoing AVR Distribution of grouped cardiac damage stages (Stage 0-1, Stage 2, and Stage 3-4). AVR = aortic valve replacement.

Stratifying patients by individual cardiac damage stages (stages 0, 1, 2, 3, and 4) following Généreux et al^3^ staging classification, 3 studies (n = 2,072) with pooled data (stage 0, n = 289; stage 1, n = 564 patients; stage 2, n = 892; and stage 3-4, n = 327) were analyzed. During a median follow-up of 4.1 years (IQR: 1.9-5.2 years), there were 617 deaths reported, resulting in a pooled crude rate of death of 33.0% (95% CI: 30.8%-35.2%). Kaplan-Meier curve analysis showed that patients with more advanced cardiac damage stages had significantly higher 1-year mortality event rates (log-rank chi-square, 75.7; *P* < 0.001) (Central Illustration). In patients who had cardiac damage classified as stage 0, 1-year mortality was 3.9%. Whereas patients classified as stage 1, 2, and 3 to 4 had a 1-year mortality of 5.7%, 9.4%, and 17.6%, respectively (1-year HR in stage 1: 1.36 [95% CI: 0.69-2.72]; *P* = 0.377, stage 2: 2.28 [95% CI: 1.21-4.28]; *P* = 0.010; and stage 3 to 4: 4.38 [95% CI: 2.29-8.36]; *P* < 0.001) (Central Illustration). At the final follow-up (8 years), mortality was 19.3%, 36.9%, 51.7%, and 67.8% in patients with cardiac damage stage 0, 1, 2, and 3-4, respectively (HR in stage 1: 1.70 [95% CI: 1.21-2.38], *P* = 0.002; stage 2: 2.20 [95% CI: 1.60-3.02]; *P* < 0.001; and stage 3 to 4: 3.90 [95% CI: 2.79-5.47]; *P* < 0.001) (Figure 4).

### Symptomatic vs asymptomatic AS

**Figure 5:**
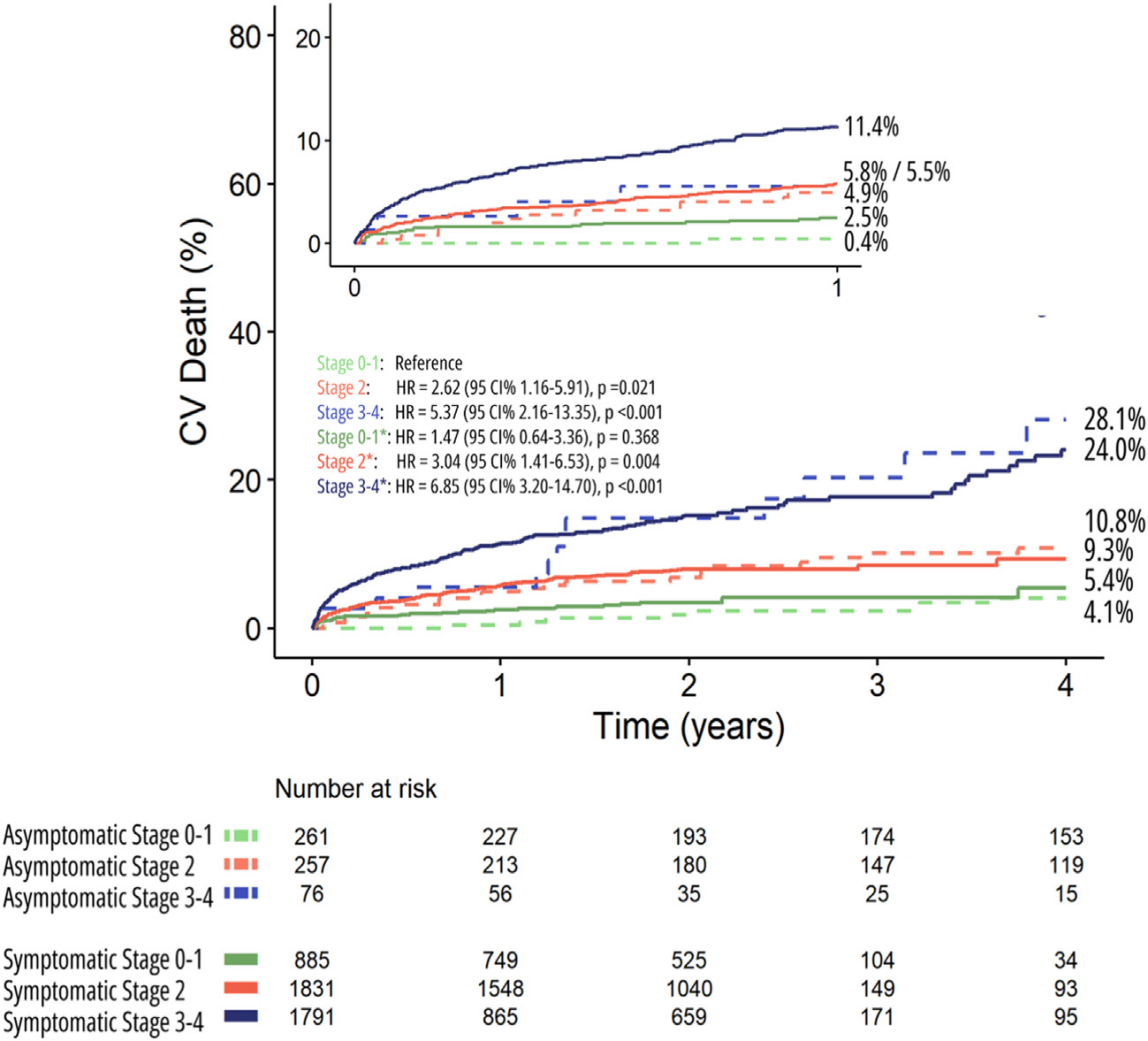
Reconstructed Kaplan-Meier Analyses for All-Cause Death in Symptomatic vs Asymptomatic Cohorts Stratified by Cardiac Damage Staging CV = cardiovascular.

To elucidate the effect of cardiac damage staging in symptomatic severe AS vs asymptomatic moderate/severe AS patients, corresponding cardiac damage stages from both reconstructed cohorts were pooled and compared, where stage 0 to 1 asymptomatic category was set as the reference cohort. By comparing reciprocal cardiac stages from both cohorts (asymptomatic vs symptomatic), we found no significant difference in HRs for all-cause mortality across all stages at 1- and 5-year follow-up (Supplemental Table 5, Figure 5).

We further assessed the differences in CV mortality as a secondary outcome between symptomatic and asymptomatic AS cohorts. CV death was significantly different between the different groups (log-rank chi-square, 219.5; *P* < 0.001) (Supplemental Table 5, Supplemental Appendix, Figure 5).

## Discussion

The current study, analyzing 14,499 patients with AS undergoing AVR, summarizes several key aspects of the prognostic impact of cardiac damage staging utilizing reconstructed pooled time-to-event patient data on mortality post-AVR. Our main findings are as follows: 1) a significant proportion of patients had extensive cardiac damage at baseline regardless of severity or symptomatic status; 2) the extent of cardiac damage was associated with all-cause and CV mortality at 5 years for symptomatic severe AS; 3) the extent of cardiac damage was associated with all-cause and CV mortality at 8 years for asymptomatic moderate and severe AS; 4) there was no difference in all-cause and CV mortality between the asymptomatic and symptomatic cohort following AVR at short- and long-term follow-up.

The current study reinforces the prognostic value of the cardiac damage staging approach in all-comers AS patients, including symptomatic and asymptomatic cohorts undergoing either surgical or transcatheter AVR. Indeed, our pooled analysis revealed that approximately 60% of asymptomatic moderate-severe AS patients who underwent AVR had evidence of cardiac damage at baseline (stage ≥2) with an approximate 2- and 4-fold increase in mortality for stages 2 and 3 to 4, respectively. These findings indicate that triggers for AVR in the current guidelines are neither specific nor sensitive in accounting for the extent of cardiac damage and subsequent mortality risk.^1^^,^^2^ Moreover, a third of the population had LV cardiac damage (stage 1), which was associated with a ∼2-fold increase in mortality risk at 8-year follow-up. Studies have shown that the cutoff of LV ejection fraction <50% in defining LV dysfunction as a trigger for AVR is not sensitive in the detection of subclinical LV dysfunction.^44^^,^^45^ Indeed, the development of cardiac damage could start long before symptom and severity manifestation of severe AS, prompting a reconsideration of more appropriate cutoffs in the timing of intervention in this population. In addition, several small-sized observational studies demonstrated a mortality benefit with AVR in patients with heart failure with reduced ejection fraction and concomitant moderate AS, with a similar benefit to those with severe symptomatic AS.^46^^,^^47^ When analyzed collectively, such observations would prompt early consideration of AVR in this population before the onset of irreversible damage. Indeed, Généreux et al^4^^,^^5^ demonstrated that the extravalvular damage resulting from AS remained unchanged or even worsened in about 85% of patients 1-year following AVR and was associated with poor outcomes, which reflects the possible unclaimed benefit of earlier intervention.

Importantly, when comparing asymptomatic to symptomatic groups, our analysis showed that there was no difference in all-cause or CV mortality in corresponding cardiac damage stages. Our findings are in line with previous studies demonstrating that the outcome of the asymptomatic moderate-severe AS population is not benign.^48^^,^^49^ These findings also underscore that symptoms are not necessarily indicative of the state of cardiac damage at the time of AVR. Moreover, the intervention in the asymptomatic moderate-severe AS cohort did not significantly impact the mortality risk across respective cardiac damage stages. These findings emphasize that the absence of symptoms might be falsely reassuring and push the needle toward preemptive early intervention to minimize the irreversible cardiac damage early on in the progressive course of AS management.

Another important finding is the heightened comparable mortality risk with advanced cardiac damage stages (stage ≥2) in both asymptomatic and symptomatic groups. Hence, the cardiac damage stage >2 might serve as a valid risk stratification tool to identify AS patients who would benefit from earlier intervention and closer echocardiographic follow-up period as opposed to the 2-year interval recommended by the guidelines.^1^^,^^2^ On the other hand, it is imperative to carefully balance the risks and benefits of early intervention and consider the most appropriate AVR intervention (surgical vs transcatheter) given the finite long-term bioprosthetic valve durability.^50^^,^^51^ Ongoing trials will bring meaningful insight into whether preemptive AVR before severe AS or symptoms develop will improve patients outcomes (severe AS and no symptoms: EARLY TAVR [Evaluation of TAVR Compared to Surveillance for Patients With Asymptomatic Severe Aortic Stenosis; NCT03042104], the EVoLVeD [Early Valve Replacement Guided by Biomarkers of LV Decompensation in Asymptomatic Patients With Severe AS; NCT03094143]) and the EASY-AS (The Early Valve Replacement in Severe ASYmptomatic Aortic Stenosis Study; NCT04204915) trials; moderate AS with evidence of cardiac damage or dysfunction: PROGRESS (Prospective, Randomized, Controlled Trial to Assess the Management of Moderate Aortic Stenosis by Clinical Surveillance or Transcatheter Aortic Valve Replacement; NCT04889872), the Evolut (Medtronic) EXPAND TAVR II Pivotal Trial; NCT05149755) and the TAVR-UNLOAD (Transcatheter Aortic Valve Replacement to UNload the Left Ventricle in Patients With ADvanced Heart Failure; NCT02661451).

### Clinical implications

Despite the current emphasis on symptomatic status in the guidelines, asymptomatic patients with moderate to severe AS had similar all-cause mortality to symptomatic severe AS within their respective cardiac damage stages. That highlights a careful consideration of earlier intervention in an effort to prevent further irreversible cardiac damage later during the lifetime course of AS. Furthermore, it would be pivotal that future studies and trials focus on the global assessment and monitoring of cardiac damage as a dynamic tool for risk stratification in determining the subset of AS patients who would benefit the most from early intervention to halt subsequent irreversible damage. Furthermore, our pooled data suggest that each of the extravalvular cardiac damage stages ensuing from AS could be perceived as individual categories for risk prediction rather than a hierarchy-based sequential progressive nature.

### Study limitations

First, very few studies accounted for confounders (such as age, sex, comorbidities, and frailty), which might lead to an inherent selection bias across cardiac damage stages. Hence, our pooled analysis is therefore unable to test the prognostic utility of stages against other variables or models. Second, despite the data supporting further echocardiographic (global longitudinal strain, stroke volume index) and biochemical markers (such as B-type natriuretic peptide, albumin) incorporated in the modified versions of the initial cardiac damage staging classification, such risk markers were not used across all the included studies, limiting a pooled analysis of such variables.^9^^,^^11^^,^^12^^,^^52^^,^^53^ Third, interval classification of cardiac damage staging during follow-up was not present in the majority of the included studies. Hence, we could not identify if the patients’ corresponding cardiac damage stage improved or worsened over follow-up. Fourth, granular individual data on background medical therapy, comorbidities, and certain factors such as the extent of CAD were not available within all the pooled studies, hindering potential insights and risk-adjusted analyses for certain factors that might impact cardiac damage extent and outcomes. Fifth, within the symptomatic cohort, the majority of studies encompassed patients who underwent AVR and were longitudinally monitored. In contrast, within the asymptomatic cohort, patient stratification based on initial AVR status was not consistently applied across all studies, with only 1 study encompassing patients who all underwent AVR immediately following cardiac damage staging. Thus, interpretation of these results in the context of an asymptomatic cohort undergoing AVR needs to be made cautiously. Nevertheless, it is noteworthy that approximately 40% of patients in the asymptomatic cohort eventually underwent AVR. Sixth, interestingly, several studies combined stage 0 to 1 and stage 3 to 4 due to the low number of patients and low power in each stage. Conceptually, it will seem more appropriate to consider stage 0 separately, pool stages 1 and 2 (LV and LA damage, representing left side damage), and stage 3 to 4 (pulmonary artery and right ventricle, representing right side damage). Indeed, patients in stage 0 have no detectable extravalvular damage yet, representing a unique population most likely at an earlier stage of the disease or more resilient to afterload mismatch compared with patients who have already established damage. Finally, the initial classification used simple criteria easily applicable by echocardiographers in clinical practice; however, the inclusion of more advanced analysis including LV strain might enhance patient risk stratification.^54^ That said, those analyses have their inherent reproducibility limitations and are not generally applicable to the general cardiology community, compromising their generalizability.

## Conclusions

In patients undergoing AVR across the symptomatic and severity spectra of AS, cardiac damage staging at baseline has important prognostic implications. This pooled meta-analysis in patients undergoing AVR suggests that staging of baseline cardiac damage could be considered for timing and selection therapy in patients with moderate or severe AS to better risk-stratify patients and determine the need for earlier AVR or adjunctive pharmacotherapy. Prospective studies and randomized trials are needed to confirm this concept.

PERSPECTIVES **COMPETENCY IN PATIENT CARE:** Cardiac damage staging has been shown to have a prognostic impact on mortality across the severity and symptom spectrum of patients with AS. Incorporating such an approach would enhance risk stratification and the timing of AVR for patients with AS.

**TRANSLATIONAL OUTLOOK:** Long-term follow-up data and prospective multicenter clinical research incorporating cardiac damage staging are expected to improve the outcomes of patients with AS undergoing AVR.

## Funding support and author disclosures

Mr Xander Jacquemyn was supported by the Belgian American Educational Foundation. Dr Généreux has served as a consultant for Abbott Vascular, Abiomed, BioTrace Medical, Boston Scientific, CARANX Medical, Cardiovascular System Inc (PI Eclipse Trial), Edwards Lifesciences (PI EARLY-TAVR trial, PI PROGRESS trial), GE Healthcare, iRhythm Technologies, Medtronic, Opsens, Pi-Cardia, Puzzle Medical, Saranas, Shockwave, Siemens, Soundbite Medical Inc, Teleflex, and 4C Medical (PI feasibility study); has served as an advisor for Abbott Vascular, Abiomed, BioTrace Medical, Edwards Lifesciences, and Medtronic; has received speaker fees from Abbott Vascular, Abiomed, BioTrace Medical, Edwards Lifesciences, Medtronic, and Shockwave; has served as a proctor for and received an institutional research grant from Edwards Lifesciences, and has equity in Pi-Cardia, Puzzle Medical, Saranas, and Soundbite Medical Inc. Dr Pibarot has received funding from Edwards Lifesciences, Medtronic, Pi-Cardia, and Cardiac Phoenix for echocardiography core laboratory analyses and research studies in the field of transcatheter valve therapies, for which he received no personal compensation, and has received lecture fees from Edwards Lifesciences and Medtronic. The Cardiovascular Research Foundation (Dr Cohen and Leon) receives research funding from Edwards Lifesciences (no direct compensation). Dr Clavel holds a young investigator grant from the QHLI. Dr Gillam has served as a consultant for Edwards Lifesciences; and has core lab contracts with Edwards Lifesciences and Medtronic. Dr Sultan received institutional research support from Abbott, Artivion, Boston Scientific, Edwards, Medtronic, and Terumo Aortic. Dr Cohen has received research grant support and consulting income from Edwards Lifesciences, Medtronic, Boston Scientific, and Abbott. Dr Bax: The Department of Cardiology (LUMC, The Netherlands) has received research grants from Medtronic, Biotronik, Edwards Lifesciences, and Boston Scientific and has received speaker fees from Abbott Vascular and Edwards Lifesciences. Dr Leon serves on the PARTNER Trial Executive Committee for Edwards Lifesciences (nonpaid); has received institutional research grants from and has served as a nonpaid advisor for Abbott, Boston Scientific, and Medtronic; has served as a nonpaid advisor for Sinomed; and has equity in Medinol. All other authors have reported that they have no relationships relevant to the contents of this paper to disclose.
