# Supplementary material for: Cardiac Damage Staging Predicts Outcomes in Aortic Valve Stenosis After Aortic Valve Replacement: Meta-Analysis
Source: JACC Adv. 2024 May 22;3(5):100959. doi: 10.1016/j.jacadv.2024.100959 (PMC11198616; doi:10.1016/j.jacadv.2024.100959)
Supplement: Supplementary data [file mmc1.docx]

**Supplemental Table 1.** Baseline Echocardiographic parameters of total population in included studies.

| **Study, Year** | **n** | **SVI, mean (SD), mL/m2** | **LVMI, mean (SD), g/m2** | **SVI <35 mL/m2** | **LVEF, mean (SD), %** | **LVEF < 35%/<50%* n(%)** | **E/e ́ ratio** | **PASP, mean (SD), mm Hg** | **LA diameter, mm** | **PW thickness, mm** | **IVS thickness, mm** | **AV gradient, mean (SD), mm Hg** | **AV mean gradient <40 mm Hg** | **AVA, mean (SD), cm2** | **AVA Index, cm2/m2** | **LAVI, mean (SD), mL/m2** | **TAPSE <1.7, mean (SD), cm** | **Moderate /severe MR n(%)** | **Moderate /severe TR, n(%)** | **Moderate /severe AR n(%)** |
| --- | --- | --- | --- | --- | --- | --- | --- | --- | --- | --- | --- | --- | --- | --- | --- | --- | --- | --- | --- | --- |
| Fukui et al., 2019 | 689 | 36.3 (11.3) | 155 (42.6) | 296 (43) | 54.2 (13) | - | - | 43.3 (16.1) | - | - | - | 47.8 (15.5) | 194 (28.2) | 0.65 (0.18) | - | 47.8 (17.2) | 208 (30.2) | 88 (12.8) | 108 (15.7) | - |
| Vollema et al., 2019 | 1,189 | - | 132.6 (39.7) | - | 54.2 (14.3) | 339 (29)* | 19.3 (10.2) | 36.5 (14) | - | 11.9 (2.2) | 12.5 (2.4) | 43.1 (15.5) | - | 0.78 (0.18) | 0.45 (0.11) | 44.5 (23.1) | 20.8 (4.4) | 68 (6) | 65 (6) |  |
| Maeder et al., 2020^#^ | 263 | - | - | - | - | - | - | - | - | - | - | - | - | - | - | - | - | - | - | - |
| Berkovitch et al., 2020 | 2608 | - | - | - | - | - | - | - | - | - | - | - | - | - | - | - | - | - | - | - |
| Avvedimento et al., 2021 | 262 | - | - | - | - | - | - | - | - | - | - | - | - | - | - | - | - | - | - | - |
| Okuno et al., 2021 | 1,133 | 82.1±6.3 |  |  |  | 557 (49.2) | - | - | - | 946 (83.5%) | 203 (17.9%) | - |  | 775 (68.5) |  |  | 162 (14.3) |  | 6.07 ± 4.2 | - |
| Schewel et al., 2021 | 1,400 | - | - | - | - | 176 (12.6) | - | - | - | - | - | - | - | - | - | - | - | 586 (41.9) | 410 (29.3) | 378 (27) |
| Hirasawa et al., 2021 | 405 | - | 90 (22) | - | 54 (15) | - | - | - | - | - | - | - | - | - | - | 62 (19) | - | - | - | - |
| Généreux et al., 2022 (PARTNER 2 &3) | 1,974 | - | - | - | - | - | - | - | - | - | - | 46.3 (13.12) | - | 0.7 (0.18) | - | - | - | - | - | - |
| Shamekhi et al., 2022 | 933 | - | 124 (100-155) |  | 56 (12.8) | - | 16 (13-22) | 33.3 (14.9) | - | - | - | - | - | - | - | 36 (16.6) | 21.1 (5.4) | 246 (26.4) | 179 (19.2) | - |
| Pellegrini et al., 2022 | 841 | - | - | - | - | - | - | - | - | - | - | - | - | - | - | - | - | - | - | - |
| Zhu et al., 2022 | 464 | - | - | - | - | - | - | - | - | - | - | - | - | - | - | - | - | - | - | - |
| Tastet et al., 2019^†^ | 735 | 42 (9) | 118 (36) | - | 63 (7) | - | 12.9 (5.7) | 33 (9) | - | - | - | 35 (16) | - | 0.94 (0.23) | 0.51 (0.13) | 38 (15) | - | 9 (1.2) | 10 (1.4) | - |
| Amanullah et al., 2021^†^ | 1,245 | - | 118.4 (34.6) | - | 58.3 (12.7) | 222 (17.8)* | 16.5 (7.3) | 36.9 (13) | - | 10.7 (1.8) | 11.2 (1.9) | 24.4 (7.6) | - | 1.2 (0.15) | - | 41.8 (22.5) | - | 123 (9.9) | 141 (11.3) | - |
| Park et al., 2021^†^ | 145 | - | - | - | - | - | - | - | - | - | - | - | - | - | - | - | - | - | - | - |
| Sevilla et al., 2023^†^ | 96 | - | 76 (20) | - | 66 (6) | - | - | 35 (10) | - | - | - | 39 (14) | - | 1.04 (0.24) | - | - | 23 (4) | 9 (9) | - | - |

**Abbreviations:** AV, aortic valve; AVA, aortic valve area; LAVI, left atrial volume index; LVEF, left ventricular ejection fraction; LVMI, left ventricular mass index; MR, mitral regurgitation; PASP, pulmonary artery systolic pressure; SVI, stroke volume index; TAPSE, tricuspid annular plane systolic excursion; TR, tricuspid regurgitation. Values are presented as frequencies n (%), or mean/median ± SD/IQR; Maeder et al., 2020^#^ , data presented according to the echocardiographic staging subgroup. ^†^ Population was moderate or severe Asymptomatic Aortic Stenosis

**Supplemental Table 2.** Baseline Characteristics of Included Studies, according to Echocardiographic Cardiac Staging

|  | **Study, Year** | **n** | **Age** | **Male** | **BMI, mean (SD)** | **DM** | **HLD** | **HTN** | **Previous Cardiac Surgery** | **MI** | **NYHA class ≥3** | **Lung Disease^a^** | **STS score** | **Afib/flutter** | **Past CVA/TIA** | **CAD** | **Renal Disease** |
| --- | --- | --- | --- | --- | --- | --- | --- | --- | --- | --- | --- | --- | --- | --- | --- | --- | --- |
| **Stage 0** | Fukui et al., 2019 | - | - | - | - | - | - | - | - | - | - | - | - | - | - | - | - |
|  | Vollema et al., 2019 | 97 | 72.7±9.9 | 65 (67) | 26.1±4.7 | 30 (31) | 67 (69) | 67 (69) | - | 12 (12) | 27 (31) | 11 (11) |  | 8 (8) | - | 42 (43) | - |
|  | Maeder et al., 2020 | 67 | 73 ±10 | 36 (54) | 27.8±4.9 | 16 (24) | - | - | - | - | 14 (21) | 9 (13) | - | 5 (7) | 7 (10) | - | - |
|  | Berkovitch et al., 2020 | 758 | 82±6.5 | 330 (44) | - | 280 (37) | 572 (76) | 638 (85) | - | - | 593 (78) | 126 (17) | 4.0±2.7 | - | 91 (12) |  |  |
|  | Avvedimento et al., 2021* | 23 | 77.3±6.8 | 9 (39.1) | 26.4 ± 4.3 | 5 (21.7) | 12 (52.2) | 19 (82.6) | - | 1 (4.3) | 5 (21.7) | 7 (30.4) | 3.5 ±1.4 | - | 2 (8.7) | 7 (30.4) | 5(21.7) |
|  | Okuno et al., 2021* | 151 | 80.5 ± 6.6 | 69 (45.7) | - | - | - | 123(81.5) | 13 (8.6) | - | 88 (58.3) | 18 (12.0) | 4.32 ± 2.70 | - | 12 (7.9) | 89 (58.9) | 91(60.3) |
|  | Snir et al., 2021 | 2,282 | - | - | - | - | - | - | - | - | - | - | - | - | - | - | - |
|  | Schewel et al., 2021 | 138 | 80.9 ± 5.9 | 70 (50.7) | 25.9±4.2 | 41(29.7) | 53 (38.4) | 114(82.6) | 12 (8.7) | - | 118 (85.5) | 25(18.1) | 5.1 [4.5–5.7] | 43 (31.2) | - | 85 (61.6) | 63 (45.7) |
|  | Hirasawa et al., 2021 | 27 | 80±6 | 12 (44) | - | 8 (30) | 19 (70) | 23 (85) | - | - | 16 (59) | 0 | - | 0 | - | 19 (70) | - |
|  | Généreux et al., 2022 (PARTNER 2 &3) | 121 | 76.6±7.88 | 75 (62) | - | 35(28.9) | - | 105 (86.8) | 13(10.7) | 8 (6.7) | 57 (47.1) | 16(13.3) | 3.3 ± 2.73 | - | 9 (7.4) | 54(44.6) | 32(26.5) |
|  | Shamekhi et al., 2022 | 43 | 79.5±6.5 | 14 (32.6) | - | 13 (30.2) | - | 37 (86) | 1 (2.3) | - | 29 (70.7) | 13 (30.2) | 3.52 ± 1.9 | 0 | - | 26 (60.5) | 18 (41.9) |
|  | Pellegrini et al., 2022 | 7 | 79.9 ± 4.0 | 3 (42.9) | 25.4 ± 3.2 | 1 (14.3) | - | 7 (100.0) | 0 (0) | - | 2 (28.6) | 1 (14.3) | - | - | 1 (14.3) | 5 (79.4) |  |
|  | Zhu et al., 2022 | 199 | 75.8 ± 6.3 | 108 (54.3) | 22.9 ± 3.4 | 48 (24.1) | 44 (22.1) | 119 (59.8) | 0 | - | 160 (80.4) | 41 (20.6) | 5.72 ± 3.77 | 0 | 9 (4.5) | - | - |
|  | Tastet et al., 2019^†^ | 109 | 64 ± 15 | 69 (63) | 27 ± 4 | 20 (18) | - | 62 (57) | 3 (3) | 11 (10) | 0 | 7 (6) | - | 0 | 5 (5) | 18 (17) | 15 (14) |
|  | Amanullah et al., 2021^†^ | 163 | 66 ± 16.5 | 106 (65) | 26.7 ± 10.4 | 39 (23.9) | 126 (77.8) | 114 (69.9) | - | 7 (4.3) | 5 (3.1) | 6 (3.7) | - | 3 (1.8) | - | 59 (36.2) | - |
|  | Park et al., 2021^†^ | 10 | - | - | - | - | - | - | - | - | - | - | - | - | - | - | - |
|  | Sevilla et al., 2023^†^ | 37 | - | - | - | - | - | - | - | - | - | - | - | - | - | - | - |
|  | | | | | | | | | | | | | | | | | |
| **Stage 1** | Fukui et al., 2019 | 93 | 81.1±7.2 | 42 (45.2) | 28.4 ±6.4 | 37 (39.8) | 73 (78.5) | 81 (87.1) | 24 (25.8) | 31 (33.3) | 69 (74.2) | 46 (49.5) | 6±4 | 0 | - | - | - |
|  | Vollema et al., 2019 | 282 | 71.6 ±11.4 | 139 (49) | 25.5 ±4.2 | 80 (28) | 185 (66) | 210 (75) | - | 36 (13) | 67 (26) | 31 (11) | - | 35 (12) | - | 131 (47) | - |
|  | Maeder et al., 2020 | 113 | 73±11 | 73 (65) | 27.6 ±4.5 | 11 (10) | - | - | - | - | 21 (19) | 15 (13) | - | 1 (1) | 4 (4) | - | - |
|  | Berkovitch et al., 2020 | 769 | 82 ±6.6 | 348 (45) | - | 287 (38) | 602 (79) | 659 (86) | - | - | 598 (78) | 107 (14) | 5 (3.3) | - | 108 (14) |  |  |
|  | Avvedimento et al., 2021* | 23 | 77.3±6.8 | 9 (39.1) | 26.4 ± 4.3 | 5 (21.7) | 12 (52.2) | 19 (82.6) | - | 1 (4.3) | 5 (21.7) | 7 (30.4) | 3.5 ± 1.4 | - | 2 (8.7) | 7 (30.4) | 5 (21.7) |
|  | Okuno et al., 2021* | 151 | 80.5 ± 6.6 | 69 (45.7) | - | - | - | 123 (81.5) | 13 (8.6) | - | 88 (58.3) | 18 (12.0) | 4.32 ± 2.70 | - | 12 (7.9) | 89 (58.9) | 91 (60.3) |
|  | Snir et al., 2021 | 1,455 | - | - | - | - | - | - | - | - | - | - | - | - | - | - | - |
|  | Schewel et al., 2021 | 330 | 81.6 ± 6.7 | 137 (41.5) | 25.8 ± 4.7 | 86 (26.1) | 113 (34.2) | 275 (83.3) | 39 (11.8) | - | 275 (83.3) | 40 (12.1) | 5.9 [5.1–6.7] | 127(38.5) | - | 203 (61.5) | 184 (55.8) |
|  | Hirasawa et al., 2021 | 96 | 78±9 | 54 (56) | - | 34 (35) | 69 (72) | 76 (79) | - | - | 57 (59) | 0 | - | 0 | - | 59 (62) | - |
|  | Généreux et al., 2022 (PARTNER 2 &3) | 287 | 77.9 ± 7.76 | 138 (48.1) | - | 102 (35.5) | - | 263 (91.6) | 33 (11.5) | 31 (10.8) | 166 (57.9) | 43 (15.1) | 4.1 ± 3.04 | - | 29 (10.1) | 117 (40.8) | 116 (40.4) |
|  | Shamekhi et al., 2022 | 462 | 80.6±6.2 | 250(54.1) | - | 125 (27.1) | - | 399 (86.3) | 42 (9.1) | - | 389 (84.2) | 83 (18) | 4.42 ± 3.3 | 147 (32) | - | 310 (67.2) | 227 (49.2) |
|  | Pellegrini et al., 2022 | 63 | 79.1 ± 5.7 | 36 (57.1) | 26.8 ± 4.6 | 23 (36.5) | - | 55 (87.3) | 3 (4.8) | - | 32 (50.8) | 7 (11.1) | - | - | 7 (11.1) | 50 (79.4) | - |
|  | Zhu et al., 2022 | 51 | 76.6 ± 7.1 | 40 (78.4) | 22.0 ± 3.4 | 8 (15.7) | 11 (21.6) | 23 (45.1) | 1 (2.0) | - | 48 (94.1) | 18 (35.3) | 9.63 ± 10.19 | 0 | 2 (3.9) | - | - |
|  | Tastet et al., 2019^†^ | 195 | 67 ± 14 | 112 (57) | 27 ± 5 | 41 (21) | - | 124 (64) | 12 (6) | 18 (9) | 0 | 21 (11) | - | 0 | 20 (10) | 34 (17) | 23 (12) |
|  | Amanullah et al., 2021^†^ | 334 | 71.5 ± 11.8 | 168 (50.3) | 25.7 ± 4.7 | 121 (36.2) | 276 (82.6) | 267 (79.9) | - | 63 (18.9) | 25 (7.5) | 27 (8.1) | - | 17 (5.1) | - | 169 (50.6) | - |
|  | Park et al., 2021^†^ | 86 | - | - | - | - | - | - | - | - | - | - | - | - | - | - | - |
|  | Sevilla et al., 2023^†^ | 32 | - | - | - | - | - | - | - | - | - | - | - | - | - | - | - |
|  | | | | | | | | | | | | | | | | | |
| **Stage 2** | Fukui et al., 2019 | 426 | 82.8±7.4 | 229 (53.8) | 28.2 (6.4) | 170 (39.9) | 336 (78.9) | 379 (89.0) | 129 (30.3) | 171 (40.1) | 338 (79.3) | 159 (37.3) | 8.2 (4.7) | 211 (49.5) | - | - | - |
|  | Vollema et al., 2019 | 588 | 73.8±10.7 | 301 (51) | 25.6 ±4.9 | 144 (25) | 397 (68) | 430 (73) | - | 85 (15) | 189 (35) | 49 (8) | - | 184 (31) | - | 267 (45) | - |
|  | Maeder et al., 2020 | 113 | 73 ± 11 | 73 (65) | 27.6 ±4.5 | 11 (10) | - | - | - | - | 21 (19) | 15 (13) | - | 1 (1) | 4 (4) | - | - |
|  | Berkovitch et al., 2020 | 730 | 82 ±6.5 | 361 (50) |  | 272 (37) | 570 (78) | 649 (89) | - | - | 599 (82) | 115 (16) | 5.4 (4) | - | 129 (18) |  |  |
|  | Avvedimento et al., 2021 | 106 | 79.2 ± 6.7 | 39 (36.8) | 27.1 ± 5.9 | 41 (38.7) | 69 (65.1) | 94 (88.7) | - | 18 (17) | 58 (54.7) | 26 (24.5) | 4.7 ± 3.1 | - | 11 (10.4) | 49 (46.2) | 34 (32.1) |
|  | Okuno et al., 2021 | 397 | 82.7 ± 5.5 | 207 (52.1) | - | - | - | 338 (85.1) | 59 (14.9) | - | 258 (65.2) | 55 (13.9) | 5.37 ± 3.38 | - | 57 (14.4) | 243 (61.2) | 270 (68.0) |
|  | Snir et al., 2021 | 3,052 | - | - | - | - | - | - | - | - | - | - | - | - | - | - | - |
|  | Schewel et al., 2021 | 469 | 81.7 ± 6.7 | 205 (43.7) | 26.9 ± 5.2 | 137 (29.2) | 187 (39.9) | 394 (84.0) | 55 (11.7) | - | 416 (88.7) | 71 (15.1) | 6.1 [5.5–6.6] | 215 (45.8) | - | 286 (61.0) | 280 (59.7) |
|  | Hirasawa et al., 2021 | 152 | 81±6 | 66 (43) | - | 38 (25) | 95 (63) | 116 (76) | - | - | 82 (54) | 32 (21) | - | 22 (15) | - | 96 (63) | - |
|  | Généreux et al., 2022 (PARTNER 2 &3) | 1,014 | 80.8 ± 7.47 | 567 (55.9) | - | 311 (30.7) | - | 929 (91.8) | 165 (16.3) | 158 (15.6) | 634 (62.5) | 229 (22.7) | 5.6 ± 3.90 | - | 144 (14.2) | 560 (55.3) | 470 (46.4) |
|  | Shamekhi et al., 2022 | 428 | 80.6 ± 6.3 | 206 (48.1) | - | 133 (31.1) | - | 373 (87.1) | 101 (23.6) | - | 371 (86.7) | 109 (25.5) | 6.2 ± 5.2 | 239 (55.8) | - | 279 (65.3) | 247 (57.7) |
|  | Pellegrini et al., 2022 | 532 | 81.1 ± 5.7 | 285 (53.6) | 27.0 ± 4.9 | 143 (26.9) | - | 491 (92.3) | 46 (8.6) | - | 346 (65.0) | 77 (14.5) | - | - | 60 (11.3) | 386 (72.6) | - |
|  | Zhu et al., 2022 | 67 | 76.5 ± 6.4 | 41 (61.2) | 23.3 ± 3.8 | 18 (26.9) | 14 (20.9) | 42 (62.7) | 0 | - | 65 (97.0) | 17 (25.4) | 7.42 ± 4.31 | 38 (56.7) | 3 (4.5) | - | - |
|  | Tastet et al., 2019^†^ | 368 | 74 ± 12 | 225 (61) | 28 ± 5 | 108 (30) | - | 283 (77) | 39 (11) | 41 (11) | - | 57 (15) | - | 120 (33) | 45 (12) | 82 (22) | 91 (25) |
|  | Amanullah et al., 2021^†^ | 530 | 71.7 ± 11.3 | 247 (46.6) | 25.9 ± 5 | 188 (35.5) | 413 (77.9) | 430 (81.1) | - | 101 (19.1) | 70 (13.2) | 30 (5.7) | - | 163 (30.8) | - | 265 (50) | - |
|  | Park et al., 2021^†^ | 49 | - | - | - | - | - | - | - | - | - | - | - | - | - | - | - |
|  | Sevilla et al., 2023^†^ | 23 | - | - | - | - | - | - | - | - | - | - | - | - | - | - | - |
|  | | | | | | | | | | | | | | | | | |
| **Stage 3** | Fukui et al., 2019 | 142 | 83.2±6.7 | 60 (42.3) | 28.0±6.9 | 61 (43.0) | 111 (78.2) | 135 (95.1) | 34 (23.9) | 46 (32.4) | 116 (81.7) | 51 (35.9) | 9.3 ±5.1 | 91 (64.1) | - | - | - |
|  | Vollema et al., 2019 | 82 | 75.0±10.3 | 34 (42) | 24.6±4.6 | 18 (22) | 49 (60) | 56 (68) | - | 14 (17) | 44 (55) | 17 (21) | - | 45 (55) | - | 30 (37) | - |
|  | Maeder et al., 2020 | 73 | 79± 8 | 32 (44) | 25.9 ± 4.3 | 16 (22) | - | - | - | - | 41 (56) | 16 (22) | - | 15 (21) | 3 (4) | - | - |
|  | Berkovitch et al., 2020 | 320 | 82±8 | 127 (40) |  | 112 (35) | 223 (71) | 265 (83) | - | - | 262 (82) | 60 (19) | 6.7 (5.2) | - | 50 (16) |  |  |
|  | Avvedimento et al., 2021 | 59 | 81.1 ± 5.2 | 18 (30.5) | 27.3 ± 5.7 | 16 (27.1) | 34 (57.6) | 51 (86.4) | - | 12 (20.3) | 34(57.6) | 11 (18.6) | 5.2 ± 3.2 | - | 3 (5.1) | 25 (42.4) | 15 (25.4) |
|  | Okuno et al., 2021 | 239 | 83.1 ± 5.7 | 84 (35.1) | - | - | - | 202 (84.5) | 29 (12.1) | - | 167 (69.9) | 38 (16.0) | 6.89 ± 5.05 | - | 29 (12.1) | 142 (59.4) | 184 (77.0) |
|  | Snir et al., 2021^#^ | 1,373 | - | - | - | - | - | - | - | - | - | - | - | - | - | - | - |
|  | Schewel et al., 2021 | 323 | 82.0 ± 6.7 | 163 (50.5) | 26.0 ± 6.0 | 109 (33.7) | 117 (36.2) | 282 (87.3) | 43 (13.3) | - | 34 (10.5) | 67 (20.7) | 8.4 [7.3–9.4] | 180 (55.7) | - | 199 (61.6) | 214 (66.3) |
|  | Hirasawa et al., 2021 | 78 | 81±6 | 52 (67) | - | 21 (27) | 52 (67) | 53 (68) | - | - | 46 (59) | 20 (26) | - | 39 (50) | - | 47 (60) | - |
|  | Généreux et al., 2022 (PARTNER 2 &3) | 412 | 83.8 ± 6.90 | 199 (48.3) | - | 113 (27.4) | - | 384 (93.2) | 82 (19.9) | 49 (11.9) | 324 (78.6) | 99 (24.2) | 7.6 ± 4.77 | - | 78 (18.9) | 254 (61.7) | 223 (54.1) |
|  | Shamekhi et al., 2022 | - | - | - | - | - | - | - | - | - | - | - | - | - | - | - | - |
|  | Pellegrini et al., 2022 | 154 | 82.2 ± 6.2 | 67 (43.5) | 25.7 ± 4.4 | 40 (26.0) | - | 141 (91.6) | 11 (7.1) | - | 118 (76.6) | 34 (22.1) | - | - | 16 (10.4) | 109 (70.8) | - |
|  | Zhu et al., 2022 | 59 | 75.3 ± 6.1 | 32 (54.2) | 21.6 ± 3.3 | 10 (16.9) | 17 (28.8) | 28 (47.5) | 0 | - | 55 (93.2) | 11 (18.6) | 8.21 ± 5.27 | 20 (33.9) | 4 (6.8) | - | - |
|  | Tastet et al., 2019^†^ | 16 | 77 ± 15 | 7 (44) | 26 ± 5 | 2 (13) | - | 13 (81) | 2 (13) | 1 (7) | 0 | 1 (6) | - | 8 (50) | 4 (25) | 4 (25) | 3 (19) |
|  | Amanullah et al., 2021^†^ | 132 | 70.9 ± 11.3 | 58 (43.9) | 23.6 ± 5 | 48 (36.4) | 104 (78.8) | 100 (75.8) | - | 21 (15.9) | 26 (19.7) | 5 (3.8) | - | 73 (55.3) | - | 54 (40.9) | - |
|  | Park et al., 2021^†^ | 0 | - | - | - | - | - | - | - | - | - | - | - | - | - | - | - |
|  | Sevilla et al., 2023^†^ | 2 | - | - | - | - | - | - | - | - | - | - | - | - | - | - | - |
|  | | | | | | | | | | | | | | | | | |
| **Stage 4** | Fukui et al., 2019 | 28 | 76.6±12.9 | 20 (71.4) | 28.2±5.4 | 15 (53.6) | 21 (75.0) | 22 (78.6) | 12 (42.9) | 15 (53.6) | 20 (71.4) | 8 (28.6) | 8.5 ±4.1 | 19 (67.9) | - | - | - |
|  | Vollema et al., 2019 | 140 | 75.3±10.2 | 85 (61) | 24.8 ±4.4 | 45 (32) | 92 (66) | 94 (67) | - | 42 (30) | 66 (49) | 21 (15) |  | 82 (59) | - | 93 (66) | - |
|  | Maeder et al., 2020 | 17 | 78±6 | 10 (59) | 29.4±5.0 | 4 (24) | - | - | - | - | 8 (47) | 1 (6) | - |  | 4 (24) | - | - |
|  | Berkovitch et al., 2020 | 31 | 80±9.5 | 18 (58) |  | 13 (43) | 23 (76) | 23 (77) | - | - | 27 (87) | 6 (20) | 5.3 (3.8) | - | 4 (13) |  |  |
|  | Avvedimento et al., 2021 | 74 | 80.4 ± 5.7 | 34 (45.9) | 28.1 ± 6 | 25 (33.8) | 44 (59.5) | 65 (87.8) | - | 16 (21.6) | 54 (73) | 27 (36.5) | 6.6 ± 4.8 | - | 12 (16.2) | 36 (48.6) | 21 (28.4) |
|  | Okuno et al., 2021 | 346 | 81.4 ± 7.2 | 197 (56.9) | - | - | - | 283 (81.8) | 102 (29.5) | - | 262 (75.9) | 51 (14.8) | 7.08 ± 4.59 | - | 37 (10.7) | 241 (69.7) | 264 (76.5) |
|  | Snir et al., 2021^#^ | 1,373 | - | - | - | - | - | - | - | - | - | - | - | - | - | - | - |
|  | Schewel et al., 2021 | 140 | 80.0 ± 8.3 | 73 (52.1) | 27.5 ± 5.3 | 51 (36.4) | 52 (37.1) | 103 (73.6) | 19 (13.6) | - | 126 (90) | 28 (20.0) | 7.2 [6.0–8.4] | 87 (62.1) | - | 88 (62.9) | 93 (66.4) |
|  | Hirasawa et al., 2021 | 52 | 80±8 | 28 (54) | - | 14 (27) | 29 (56) | 41 (79) | - | - | 34 (66) | 13 (25) | - | 19 (37) | - | 25 (48) | - |
|  | Généreux et al., 2022 (PARTNER 2 &3) | 140 | 79.3 ± 8.35 | 107 (76.4) | - | 65 (46.4) | - | 123 (87.9) | 56 (40) | 38 (27.1) | 118 (84.3) | 47 (33.6) | 7.4 ± 4.74 | - | 23 (16.4) | 98 (70) | 83 (59.3) |
|  | Shamekhi et al., 2022 | - | - | - | - | - | - | - | - | - | - | - | - | - | - | - | - |
|  | Pellegrini et al., 2022 | 85 | 81.0 ± 5.9 | 52(61.2) | 26.7 ± 4.5 | 33 (38.8) | - | 70 (82.4) | 23 (27.1) | - | 70 (82.4) | 14 (16.5) | - | - | 10 (11.8) | 67 (78.8) | - |
|  | Zhu et al., 2022 | 51 | 77.3 ± 7.5 | 28 (54.9) | 22.5 ± 3.5 | 13 (25.5) | 7 (13.7) | 21 (41.2) | 1 (2.0) | - | 49 (96.1) | 12 (23.5) | 8.93 ± 5.62 | 14 (27.5) | 4 (7.8) | - | - |
|  | Tastet et al., 2019^†^ | 47 | 75 ± 14 | 29 (62) | 26 ± 5 | 10 (21) | - | 34 (72) | 6 (13) | 6 (13) | 0 | 10 (21) | - | 24 (51) | 5 (11) | 11 (23) | 15 (32) |
|  | Amanullah et al., 2021^†^ | 86 | 72.9 ± 10.6 | 43 (50) | 23.5 ± 4 | 39 (45.3) | 79 (91.9) | 75 (87.2) | - | 23 (26.7) | 19 (22.1) | 2 (2.3) | - | 42 (48.8) | - | 51 (59.3) | - |
|  | Park et al., 2021^†^ | 0 | - | - | - | - | - | - | - | - | - | - | - | - | - | - | - |
|  | Sevilla et al., 2023^†^ | 2 | - | - | - | - | - | - | - | - | - | - | - | - | - | - | - |

*Stage 0 and Stage 1 grouped in corresponding studies, **^#^**Stage 3 and Stage 4 are grouped in corresponding studies. **Abbreviations:** Afib, atrial fibrillation; BMI, body mass index (calculated as weight in kilograms divided by height in meters squared); CABG, coronary artery bypass graft; HLD, Dyslipidemia/Hyperlipidemia; CHF, congestive heart failure; MI, myocardial infarction; NYHA, New York Heart Association. Values are presented as frequencies n (%), or mean/median ± SD/IQR, † Population was moderate or severe Asymptomatic Aortic Stenosis

**Supplemental Table 3.** Baseline Echocardiographic Parameters of Included Studies, according to Echocardiographic Cardiac Staging

|  | **Study, Year** | **n** | **SVI, mean (SD), mL/m2** | **LVMI, mean (SD), g/m2** | **SVI <35 mL/m2** | **LVEF, mean (SD), %** | **LVEF < 35%** | **E/e ́ ratio** | **PASP, mean (SD), mm Hg** | **LA diameter, mm** | **PW thickness, mm** | **IVS thickness, mm** | **AV gradient, mean (SD), mm Hg** | **AV mean gradient <40 mm Hg** | **AVA, mean (SD), cm2** | | **AVA Index, cm2/m2** | **LAVI, mean (SD), mL/m2** | **TAPSE <1.7, mean (SD), cm** | **Moderate /severe MR** | **Moderate /severe TR** | **Moderate /severe AR** |  |
| --- | --- | --- | --- | --- | --- | --- | --- | --- | --- | --- | --- | --- | --- | --- | --- | --- | --- | --- | --- | --- | --- | --- | --- |
| **Stage 0** | Fukui et al., 2019 | - | - | - | - | - | - | - | - | - | - | - | - | - | - | | - | - | - | - | - | - |  |
|  | Vollema et al., 2019 | 97 | - | 87.7 (14.5) | - | 62.9 (7) | 0 | 10.8 (2.2) | 26.9 (8.7) | - | 10.9 (1.4) | 11.4 (1.5) | 41.9 (12.5) | - | 0.84 (0.19) | | 0.47 (0.11) | 24.8 (5.9) | 22.2 (3.3) | 0 | 0 | - |  |
|  | Maeder et al., 2020^#^ | 76 | 38 (9) | - | - | 63 (7) | 0 | 10 (2) | 34 (9) | - | - | - | 50 (17) | - | 0.83 (0.24) | | 0.41 (0.12) | 28 (4) | 24 (4) | 0 | - | 4 (5) |  |
|  | Berkovitch et al., 2020 | 758 | - | - | - | - | - | - | - | - | - | - | - | - | - | | - | - | - | - | - | - |  |
|  | Avvedimento et al., 2021* | 23 | - | - | - | - | - | - | - | - | - | - | - | - | - | | - | - | - | - | - | - |  |
|  | Okuno et al., 2021* | 151 | - | - | - | - | - | - | - | - | - | - | - | - | - | | - | - | - | - | - | - |  |
|  | Schewel et al., 2021 | 138 | - | 144.2 (41) | - | 56.4 (9.2) | 3 (2.2) | 12.7 (4.5) | 35.2 (9.2) | 44.4 (6.6) | 13.3 (2.6) | 13.9 (4) | 39.9 (12.1) | - | 0.83 (.22) | | 0.45 (0.12) | - | 21.1 (4.7) | 32 (23.2) | 20 (14.5) | 27 (19.6) |  |
|  | Hirasawa et al., 2021 | 27 | - | 65 (14) | - | 62 (7) | - | - | - | - | - | - | - | - | - | | - | 45 (6) | - | - | - | - |  |
|  | Généreux et al., 2022 (PARTNER 2 &3) | 121 | - | - | - | - | - | - | - | - | - | - | 45.2 (10.11) | - | 0.8 (0.14) | | - | - | - | - | - | - |  |
|  | Shamekhi et al., 2022 | 43 | - | - | - | - | - | - | - | - | - | - | - | - | - | | - | - | - | - | - | - |  |
|  | Pellegrini et al., 2022 | 7 | - | - | - | - | - | - | - | - | - | - | - | - | - | | - | - | - | - | - | - |  |
|  | Zhu et al., 2022 | 199 | - | - | - | - | - | - | - | - | - | - | - | - | - | | - | - | - | - | - | - |  |
|  | Tastet et al., 2019^†^ | 109 | 40 (7) | 90 (14) | - | 66 (4) | - | 9.4 (2.1) | 31 (6) | - | - | - | 30 (14) | - | 0.98 (0.24) | | 0.52 (0.14) | 26 (5) | - | 0 | 0 | - |  |
|  | Amanullah et al., 2021^†^ | 163 | - | 88.4 (15.1) | - | 64.9 (6.4) | 0* | 10.3 (2.2) | 29.2 (6.8) | - | 9.9 (1.5) | 10.5 (1.6) | 26 (7) | - | 1.23 (0.16) | | - | 25.9 (5.3) | - | 0 | 0 | - |  |
|  | Park et al., 2021^†^ | 10 | - | - | - | - | - | - | - | - | - | - | - | - | - | | - | - | - | - | - | - |  |
|  | Sevilla et al., 2023^†^ | 37 | - | - | - | - | - | - | - | - | - | - | - | - | - | | - | - | - | - | - | - |  |
|  | | | | | | | | | | | | | | | | | | | | | | | |
| **Stage 1** | Fukui et al., 2019 | 93 | 37.3 (9.7) | 141 (36.4) | 33 (35.5) | 58.1 (10.6) | - | - | 43.3 (16.1) | - | - | - | 50.7 (17) | 20 (21.5) | | 0.67 (0.17) | - | 27.5 (5) | 18 (19.4) | 0 | 0 | - |  |
|  | Vollema et al., 2019 | 282 | - | 124.5 (30) | - | 57.8 (12) | 52 (18)* | 18 (8) | 30.4 (8.5) | - | 11.7 (1.8) | 12.3 (1.9) | 43.9 (14.4) | - | | 0.78 (0.17) | 0.45 (0.1) | 26.1 (6.1) | 21.9 (3.5) | 0 | 0 | - |  |
|  | Maeder et al., 2020^#^ | 62 | 35 (10) | - | - | 51 (12) | 29 (47)* | 19 (5) | 35 (9) | - | - | - | 45 (15) | - | | 0.8 (0.21) | 0.4 (0.1) | 29 (4) | 22 (4) | 0 | - | 8 (13) |  |
|  | Berkovitch et al., 2020 | 769 | - | - | - | - | - | - | - | - | - | - | - | - | | - | - | - | - | - | - | - |  |
|  | Avvedimento et al., 2021* | 23 | - | - | - | - | - | - | - | - | - | - | - | - | | - | - | - | - | - | - | - |  |
|  | Okuno et al., 2021* | 151 | - | - | - | - | - | - | - | - | - | - | - | - | | - | - | - | - | - | - | - |  |
|  | Schewel et al., 2021 | 330 | - | 139 (37) | - | 55.8 (9.7) | 22 (6.7) | 12.7 (4.5) | 35 (9.9) | 45.3 (6.6) | 13.3 (2.8) | 13.8 (2.3) | 41 (14.2) | - | | 0.78 (0.21) | 0.43 (0.12) | - | 19.3 (4.6) | 103 (31.2) | 61 (18.5) | 76 (23) |  |
|  | Hirasawa et al., 2021 | 96 | - | 90 (18) | - | 57 (15) | - | - | - | - | - | - | - | - | | - | - | 47 (7) | - | - | - | - |  |
|  | Généreux et al., 2022 (PARTNER 2 &3) | 287 | - | - | - | - | - | - | - | - | - | - | 46.5 (12.26) | - | | 0.7 (0.16) | - | - | - | - | - | - |  |
|  | Shamekhi et al., 2022 | 462 | - | - | - | - | - | - | - | - | - | - | - | - | | - | - | - | - | - | - | - |  |
|  | Pellegrini et al., 2022 | 63 | - | - | - | - | - | - | - | - | - | - | - | - | | - | - | - | - | - | - | - |  |
|  | Zhu et al., 2022 | 51 | - | - | - | - | - | - | - | - | - | - | - | - | | - | - | - | - | - | - | - |  |
|  | Tastet et al., 2019^†^ | 195 | 43 (9) | 122 (31) | - | 62 (7) | - | 12.5 (5) | 30 (7) | - | - | - | 35 (16) | - | | 0.95 (0.23) | 0.52 (0.13) | 27 (5) | - | 0 | 0 | - |  |
|  | Amanullah et al., 2021^†^ | 334 | - | 115.1 (26.8) | - | 60.1 (11.5) | 47 (14.1)* | 15.1 (5.6) | 32.1 (8.5) | - | 10.9 (1.6) | 11.5 (2) | 25.6 (7.8) | - | | 1.2 (0.16) | - | 27.7 (4.7) | - | 0 | 0 | - |  |
|  | Park et al., 2021^†^ | 86 | - | - | - | - | - | - | - | - | - | - | - | - | | - | - | - | - | - | - | - |  |
|  | Sevilla et al., 2023^†^ | 32 | - | - | - | - | - | - | - | - | - | - | - | - | | - | - | - | - | - | - | - |  |
|  | | | | | | | | | | | | | | | | | | | | | | | |
| **Stage 2** | Fukui et al., 2019 | 426 | 37.4 (10.6) | 157 (42.4) | 169 (39.7) | 55.4 (11.6) | - | - | 37.9 (10.9) | - | - | - | 48.4 (15.2) | 115 (27) | | 0.66 (0.18) | - | 49.6 (15.4) | 110 (25.8) | 49 (11.5) | 0 | - |  |
|  | Vollema et al., 2019 | 588 | - | 140.7 (42.4) | - | 55.1 (13.4) | - | 19.8 (10.3) | 34.9 (10) | - | 12.2 (2.3) | 12.9 (2.6) | 46 (16) | - | | 0.78 (0.18) | 0.46 (0.18) | 50.8 (19.1) | 21.8 (3.6) | 35 (6) | 0 | - |  |
|  | Maeder et al., 2020^#^ | 113 | 32 (12) | - | - | 53 (13) | 21 (25)* | 19 (8) | 38 (8) | - | - | - | 43 (19) | - | | 0.83 (0.28) | 0.43 (0.14) | 50 (16) | 22 (4) | 29 (35) | - | 11 (13) |  |
|  | Berkovitch et al., 2020 | 730 | - | - | - | - | - | - | - | - | - | - | - | - | | - | - | - | - | - | - | - |  |
|  | Avvedimento et al., 2021 | 106 | - | - | - | - | - | - | - | - | - | - | - | - | | - | - | - | - | - | - | - |  |
|  | Okuno et al., 2021 | 397 | - | - | - | - | - | - | - | - | - | - | - | - | | - | - | - | - | - | - | - |  |
|  | Schewel et al., 2021 | 469 | - | 148.3 (46.8) | - | 53.2 (11.8) | 45 (9.6) | 15 (6.4) | 42.5 (10.4) | 46.1 (6.1) | 13.2 (2.7) | 13.9 (3.4) | 41.1 (15) | - | | 0.77 (0.2) | 0.42 (0.11) | - | 19.1 (4.6) | 193 (41.2) | 125 (26.7) | 141 (24.3) |  |
|  | Hirasawa et al., 2021 | 152 | - | 92 (22) | - | 57 (14) | - | - | - | - | - | - | - | - | | - | - | 65 (11) | - | - | - | - |  |
|  | Généreux et al., 2022 (PARTNER 2 &3) | 1,014 | - | - | - | - | - | - | - | - | - | - | 47.5 (12.96) | - | | 0.7 (0.17) | - | - | - | - | - | - |  |
|  | Shamekhi et al., 2022 | 428 | - | - | - | - | - | - | - | - | - | - | - | - | | - | - | - | - | - | - | - |  |
|  | Pellegrini et al., 2022 | 532 | - | - | - | - | - | - | - | - | - | - | - | - | | - | - | - | - | - | - | - |  |
|  | Zhu et al., 2022 | 67 | - | - | - | - | - | - | - | - | - | - | - | - | | - | - | - | - | - | - | - |  |
|  | Tastet et al., 2019^†^ | 368 | 43 (10) | 125 (39) | - | 63 (7) | - | 13.9 (6.4) | 34 (8) | - | - | - | 36 (16) | - | | 0.94 (0.23) | 0.50 (0.13) | 45 (13) | - | 6 (2) | 0 | - |  |
|  | Amanullah et al., 2021^†^ | 530 | - | 125.9 (35.2) | - | 58.1 (12.2) | 95 (17.9) | 18.2 (7.7) | 35 (9.3) | - | 11 (1.8) | 11.5 (1.9) | 24.5 (7) | - | | 1.22 (0.15) | - | 45.9 (15.4) | - | 62 (11.7) | 0 | - |  |
|  | Park et al., 2021^†^ | 49 | - | - | - | - | - | - | - | - | - | - | - | - | | - | - | - | - | - | - | - |  |
|  | Sevilla et al., 2023^†^ | 23 | - | - | - | - | - | - | - | - | - | - | - | - | | - | - | - | - | - | - | - |  |
|  | | | | | | | | | | | | | | | | | | | | | | | |
| **Stage 3** | Fukui et al., 2019 | 142 | 34.4 (13.1) | 157 (47.1) | 71 (50) | 51.8 (14.3) | - | - | 61.2 (16.4) | - | - | - | 46.3 (15.1) | 43 (30.3) | | 0.61 (0.18) | - | 54.3 (19) | 57 (40.1) | 29 (20.4) | 94 (66.2) | - |  |
|  | Vollema et al., 2019 | 82 | - | 142.3 (36.6) | - | 46.9 (14.9) | 39 (48)* | 24.2 (11.4) | 61.4 (14.6) | - | 11.8 (2) | 12.2 (2.3) | 38.2 (14.1) | - | | 0.75 (0.2) | 0.45 (0.12) | 60.4 (34.3) | 20.1 (3.6) | 14 (17) | 39 (48) | - |  |
|  | Maeder et al., 2020^#$^ | 42 | 29 (10) | - | - | 46 (15) | 22 (52)* | 26 (14) | 59 (14) | - | - | - | 40 (17) | - | | 0.79 (0.23) | 0.41 (0.13) | 61 (20) | 15 (4) | 13 (31) | - | 4 (10) |  |
|  | Berkovitch et al., 2020 | 320 | - | - | - | - | - | - | - | - | - | - | - | - | | - | - | - | - | - | - | - |  |
|  | Avvedimento et al., 2021 | 59 | - | - | - | - | - | - | - | - | - | - | - | - | | - | - | - | - | - | - | - |  |
|  | Okuno et al., 2021 | 239 | - | - | - | - | - | - | - | - | - | - | - | - | | - | - | - | - | - | - | - |  |
|  | Schewel et al., 2021 | 323 | - | 148.4 (43.5) | - | 48.7 (13.9) | 64 (19.8) | 16.6 (7.3) | 54.7 (15.3) | 47.8 (6.5) | 13.1 (3.8) | 13.6 (3.5) | 38.3 (15.6) | - | | 0.74 (0.23) | 0.41 (0.13) | - | 16.8 (4.6) | 169 (52.3) | 129 (39.9) | 94 (29.1) |  |
|  | Hirasawa et al., 2021 | 78 | - | 92 (23) | - | 53 (13) | - | - | - | - | - | - | - | - | | - | - | 75 (27) | - | - | - | - |  |
|  | Généreux et al., 2022 (PARTNER 2 &3) | 412 | - | - | - | - | - | - | - | - | - | - | 46.1 (14.68) | - | | 0.6 (0.19) | - | - | - | - | - | - |  |
|  | Shamekhi et al., 2022 | - | - | - | - | - | - | - | - | - | - | - | - | - | | - | - | - | - | - | - | - |  |
|  | Pellegrini et al., 2022 | 154 | - | - | - | - | - | - | - | - | - | - | - | - | | - | - | - | - | - | - | - |  |
|  | Zhu et al., 2022 | 59 | - | - | - | - | - | - | - | - | - | - | - | - | | - | - | - | - | - | - | - |  |
|  | Tastet et al., 2019^†^ | 16 | 38 (5) | 110 (29) | - | 64 (8) | - | 15.5 (6.5) | 54 (16) | - | - | - | 28 (14) | - | | 0.90 (0.22) | 0.51 (0.12) | 56 (37) | - | 1 (6) | 8 (50) | - |  |
|  | Amanullah et al., 2021^†^ | 132 | - | 127.7 (43.2) | - | 54.2 (14.2) | 36 (27.3)* | 19.5 (8.5) | 52.6 (15.3) | - | 10.6 (2.1) | 10.9 (2.1) | 23 (7.3) | - | | 1.17 (0.14) | - | 66.6 (42.3) | - | 42 (31.8) | 110 (83.3) | - |  |
|  | Park et al., 2021^†^ | 0 | - | - | - | - | - | - | - | - | - | - | - | - | | - | - | - | - | - | - | - |  |
|  | Sevilla et al., 2023^†^ | 2 | - | - | - | - | - | - | - | - | - | - | - | - | | - | - | - | - | - | - | - |  |
|  | | | | | | | | | | | | | | | | | | | | | | | |
| **Stage 4** | Fukui et al., 2019 | 28 | 23.3 (7.9) | 163 (30.4) | 23 (82.1) | 31.8 (13.3) | - | - | 54.7 (19.2) | - | - | - | 35.8 (10.7) | 16 (57.1) | | 0.56 (0.16) | - | 53.0 (14.8) | 23 (82.1) | 10 (35.7) | 14 (50) | - |  |
|  | Vollema et al., 2019 | 140 | - | 138.2 (34.9) | - | 41.6 (16.1) | 92 (66)* | 23.3 (12.7) | 42.8 (16.6) | - | 11.5 (2.3) | 12.3 (2.5) | 33.5 (14.3) | - | | 0.73 (0.17) | 0.43 (0.12) | 57.9 (28.2) | 13.3 (1.9) | 19 (14) | 26 (19) | - |  |
|  | Maeder et al., 2020^#^ | 42 | 29 (10) | - | - | 46 (15) | 22 (52)* | 26 (14) | 59 (14) | - | - | - | 40 (17) | - | | 0.79 (0.23) | 0.41 (0.13) | 61 (20) | 15 (4) | 13 (31) | - | 4 (10) |  |
|  | Berkovitch et al., 2020 | 31 | - | - | - | - | - | - | - | - | - | - | - | - | | - | - | - | - | - | - | - |  |
|  | Avvedimento et al., 2021 | 74 | - | - | - | - | - | - | - | - | - | - | - | - | | - | - | - | - | - | - | - |  |
|  | Okuno et al., 2021 | 346 | - | - | - | - | - | - | - | - | - | - | - | - | | - | - | - | - | - | - | - |  |
|  | Schewel et al., 2021 | 140 | - | 143 (35.8) | - | 44 (14.7) | 42 (30) | 16.6 (6.7) | 61.3 (15.2) | 47.8 (5.6) | 12.6 (2.7) | 13 (2.5) | 37.4 (17.7) | - | | 0.7 (0.22) | 0.38 (0.12) | - | 15.3 (4.1) | 89 (63.6) | 75 (53.6) | 40 (28.6) |  |
|  | Hirasawa et al., 2021 | 52 | - | 96 (26) | - | 39 (18) | - | - | - | - | - | - | - | - | | - | - | 71 (20) | - | - | - | - |  |
|  | Généreux et al., 2022 (PARTNER 2 &3) | 140 | - | - | - | - | - | - | - | - | - | - | 39.1 (10.92) | - | | 0.7 (0.22) | - | - | - | - | - | - |  |
|  | Shamekhi et al., 2022 | - | - | - | - | - | - | - | - | - | - | - | - | - | | - | - | - | - | - | - | - |  |
|  | Pellegrini et al., 2022 | 85 | - | - | - | - | - | - | - | - | - | - | - | - | | - | - | - | - | - | - | - |  |
|  | Zhu et al., 2022 | 51 | - | - | - | - | - | - | - | - | - | - | - | - | | - | - | - | - | - | - | - |  |
|  | Tastet et al., 2019^†^ | 47 | 40 (13) | 121 (38) | - | 62 (8) | - | 14.6 (5.9) | 38 (13) | - | - | - | 35 (17) | - | | 0.88 (0.24) | 0.49 (0.14) | 48 (16) | - | 2 (4) | 2 (4) | - |  |
|  | Amanullah et al., 2021^†^ | 86 | - | 125.1 (36.7) | - | 46.1 (16.1) | 44 (51.2)* | 21.1 (7.5) | 45.1 (17.6) | - | 10.5 (1.7) | 10.7 (1.7) | 17.9 (7.6) | - | | 1.19 (0.14) | - | 54 (22.6) | - | 19 (22.1) | 31 (36) | - |  |
|  | Park et al., 2021^†^ | 0 | - | - | - | - | - | - | - | - | - | - | - | - | | - | - | - | - | - | - | - |  |
|  | Sevilla et al., 2023^†^ | 2 | - | - | - | - | - | - | - | - | - | - | - | - | | - | - | - | - | - | - | - |  |

*Stage 0 and Stage 1 grouped in corresponding studies, **^#^**Stage 3 and Stage 4 are grouped in corresponding studies. ^†^ Population was moderate or severe Asymptomatic Aortic Stenosis, **Abbreviations:** AV, aortic valve; AVA, aortic valve area; LAVI, left atrial volume index; LVEF, left ventricular ejection fraction; LVMI, left ventricular mass index; MR, mitral regurgitation; PASP, pulmonary artery systolic pressure; SVI, stroke volume index; TAPSE, tricuspid annular plane systolic excursion; TR, tricuspid regurgitation. Values are presented as frequencies n (%), or mean/median ± SD/IQR; Maeder et al., 2020^#^ , data presented according to the echocardiographic staging subgroup., $: Group 3 and 4 grouped.

**Supplemental Table 4.** Pooled Estimates of Characteristics in Symptomatic Severe AS, according to Cardiac Damage Stage [0-1, 2, 3-4].

|  | **Stage 0-1, N = 3,733** | | **Stage 2, N = 4,885** | | **Stage 3-4, N = 3,664** | |
| --- | --- | --- | --- | --- | --- | --- |
| **Variable** | **Proportion / Mean (95% CI)** | ***I^2^, p-value*** | **Proportion / Mean (95% CI)** | ***I^2^, p-value*** | **Proportion / Mean (95% CI)** | ***I^2^, p-value*** |
| Age, years | 78.1 (76.7-79.5) | 96.7%, <0.001 | 79.7 (77.8-81.5) | 97.6%, <0.001 | 80 (78.9-81.1) | 92.5%, <0.001 |
| Male, % | 51.7% (48-55.5) | 80.3%, <0.001 | 51.0% (47.9-54.1) | 77.8%, <0.001 | 51.6% (46.7-56.5) | 86%, <0.001 |
| DM, % | 28.7% (24.8-32.5) | 83.7%, <0.001 | 29.1% (24.6-33.5) | 90.5%, <0.001 | 31.6% (28.1-35) | 68.1%, <0.001 |
| HLD, % | 56.5% (43.9-69.1) | 98.1%, <0.001 | 59.3% (46-72.5) | 98.1%, <0.001 | 55.4% (44.7-66.0) | 95.1%, <0.001 |
| HTN, % | 81.3% (77.9-84.8) | 86.9%, <0.001 | 84.7% (81.2-88.3) | 92.7%, <0.001 | 79.4% (75.1-83.7) | 91.1%, <0.001 |
| Previous Cardiac Surgery, % | 8.6% (5.8-11.5) | 76.7%, <0.001 | 17.4% (11.9-22.9) | 94.9%, <0.001 | 19.8% (13.5-26) | 93.8%, <0.001 |
| MI, % | 12.6% (7.4-17.7) | 82.2%, <0.001 | 21.7% (11.6-31.9) | 96.9%, <0.001 | 25.3% (17.6-32.9) | 88.0%, <0.001 |
| NYHA class ≥3, % | 57.9% (48.4-67.5) | 98.2%, <0.001 | 65.6% (54.1-77.2) | 99.0%, <0.001 | 70.1% (58.1-82.1) | 98.7%, <0.001 |
| Lung Disease, % | 16.9% (14.2-19.6) | 78.0%, <0.001 | 19.5% (15.1-23.8) | 94.0%, <0.001 | 21.4% (18.7-24.1) | 67.3%, <0.001 |
| Pre-TAVR Afib/flutter, % | 18.5% (6.6-30.5) | 98.3%, <0.001 | 36.2% (16.2-56.2) | 99.4%, <0.001 | 48.4% (39.5-57.3) | 89.6%, <0.001 |
| Past CVA/TIA, % | 8.6% (6-11.1) | 76.4%, <0.001 | 11.1% (7.3-14.8) | 89.1%, <0.001 | 11.6% (8.9-14.3) | 69.4%, <0.001 |
| CAD, % | 56.5% (49.9-63.2) | 88.7%, <0.001 | 59% (52.4-65.5) | 93.9%, <0.001 | 60.8% (55.9-65.7) | 83.0%, <0.001 |
| CKD, % | 43.8% (36-51.6) | 88.9%, <0.001 | 53.2% (43.4-63.0) | 95.6%, <0.001 | 57.3% (46.5-68.0) | 95.7%, <0.001 |
| BMI, kg/m2 | 25.9 (24.8-26.9) | 95.3%, <0.001 | 26.5 (25.4-27.7) | 94.7%, <0.001 | 26.1 (24.9-27.2) | 93.9%, <0.001 |
| STS-score, % | 4.7 (4.1-5.3) | 97.4%, <0.001 | 6.2 (5.3-7.1) | 95.9%, <0.001 | 7.4 (6.7-8.0) | 92.0%, <0.001 |
| LVMI, g/m2 | 113 (89.5-136.5) | 99.4%, <0.001 | 134.5 (106-163) | 99.6%, <0.001 | 134.9 (116.4-153.3) | 98.5%, <0.001 |
| LVEF, % | 58.3 (55.8-60.8) | 94.1%, <0.001 | 54.7 (53.4-56) | 74.4%, 0.004 | 45 (40.8-49.2) | 92.5%, <0.001 |
| PASP, mmHg | 34.1 (30.5-37.8) | 95.6%, <0.001 | 38.3 (35.2-41.4) | 97.9%, <0.001 | 56.5 (51.3-61.6) | 95.6%, <0.001 |
| AV gradient, mmHg | 44.7 (42.3-47.1) | 88.8%, <0.001 | 45.3 (42.6-48.1) | 94.8%, <0.001 | 39.5 (36.6-42.4) | 94.1%, <0.001 |
| AVA, cm2 | 0.8 (0.7-0.8) | 93.5%, <0.001 | 0.7 (0.7-0.8) | 97.7%, <0.001 | 0.7 (0.6-0.7) | 95.1%, <0.001 |
| LAVI, mL/m2 | 32.5 (25.6-39.4) | 99.4%, <0.001 | 53.9 (46.5-61.2) | 98.6%, <0.001 | 61.7 (55.5-67.8) | 89.9%, <0.001 |

**Abbreviations**: Afib, atrial fibrillation; AV, aortic valve; AVA, aortic valve area; BMI, body mass index (calculated as weight in kilograms divided by height in meters squared); CAD, coronary artery disease; CKD, chronic kidney disease; CVA/TIA, cerebrovascular accident /transient ischemic attack; DM, diabetes mellitus; HLD, dyslipidemia/hyperlipidemia; HTN, hypertension; LAVI, left atrial volume index; LVEF, left ventricular ejection fraction; LVMI, left ventricular mass index; MI, myocardial infarction; NYHA, New York Heart Association; PASP, pulmonary artery systolic pressure; STS, Society of Thoracic Surgeons Predicted Risk of Mortality. Values are presented as % or mean (95% CI).

**Supplemental Table 5.** Comparison of Hazard Ratio for All-cause Mortality and Cardiovascular Mortality between Symptomatic Severe Aortic Stenosis and asymptomatic Moderate/Severe Aortic Stenosis Cohorts.

| Asymptomatic vs. Symptomatic | HR (95% CI) | p-value |
| --- | --- | --- |
| **All-Cause Mortality** | | |
| **1-year follow-up** | | |
| Stage 0-1 | 1.75 (1.14-2.68) | **0.011** |
| Stage 2 | 1.31 (0.93-1.85) | 0.128 |
| Stage 3-4 | 1.36 (0.92-2.00) | 0.125 |
| **5-year follow-up** | | |
| Stage 0-1 | 1.15 (0.88-1.49) | 0.299 |
| Stage 2 | 1.16 (0.92-1.45) | 0.208 |
| Stage 3-4 | 1.22 (0.94-1.58) | 0.133 |
| **Cardiovascular Mortality** | | |
| **1-year follow-up** | | |
| Stage 0-1 | 6.39 (0.82-50.0) | 0.077 |
| Stage 2 | 1.20 (0.63-2.28) | 0.582 |
| Stage 3-4 | 2.35 (0.86-6.38) | 0.095 |
| **4-year follow-up** | | |
| Stage 0-1 | 1.47 (0.64-3.36) | 0.368 |
| Stage 2 | 1.16 (0.72-1.87) | 0.543 |
| Stage 3-4 | 1.28 (0.71-2.28) | 0.410 |

Reference group was asymptomatic moderate/severe Aortic Stenosis.

**Supplemental Figure 1.** PRISMA Flow Diagram for Study Selection


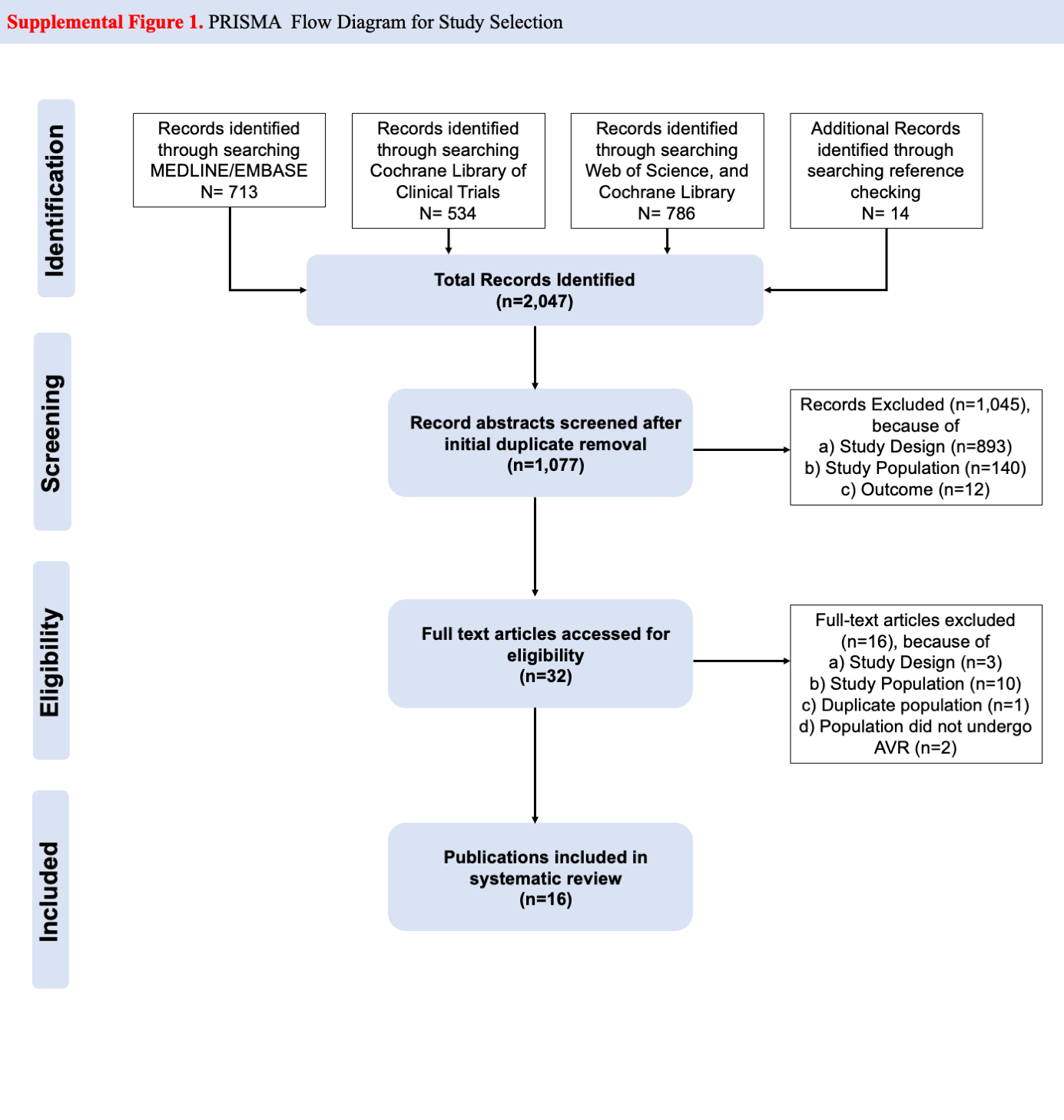


**Supplemental Figure 2**. Risk of Bias Assessment using ROBINS-E tool


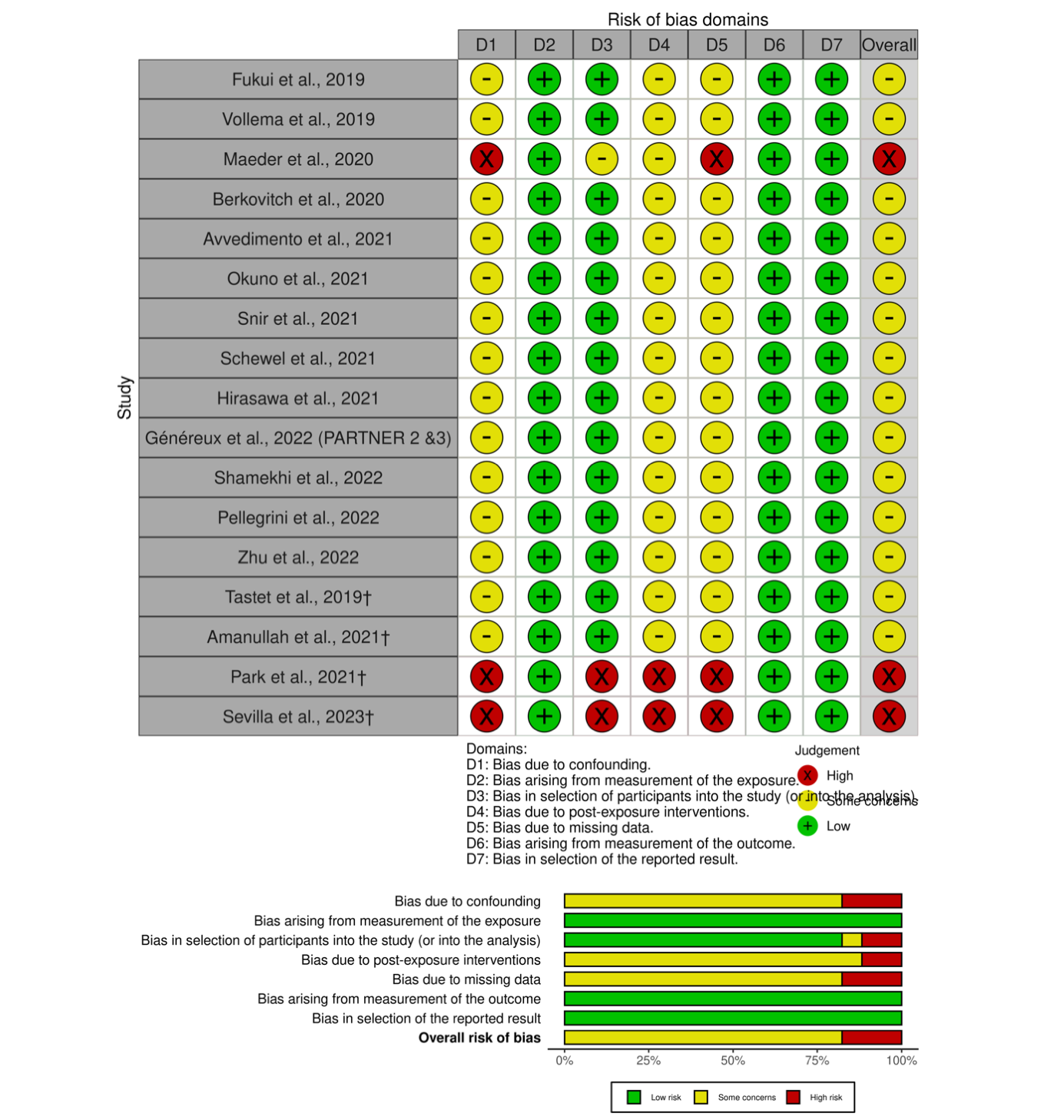


**Footnote:** ROBINS-E tool assessed risk of bias based on seven domains; Domain 1: Risk of bias due to confounding, Domain 2: Risk of bias arising from measurement of the exposure, Domain 3: Risk of bias in selection of participants into the study (or into the analysis), Domain 4: Risk of bias due to post-exposure interventions, Domain 5: Risk of bias due to missing data, Domain 6: Risk of bias arising from measurement of the outcome, Domain 7: Risk of bias in selection of the reported result ^1^

**Supplemental Figure 3.** Forest Plot for Stroke in severe symptomatic aortic stenosis according to cardiac damage stages.


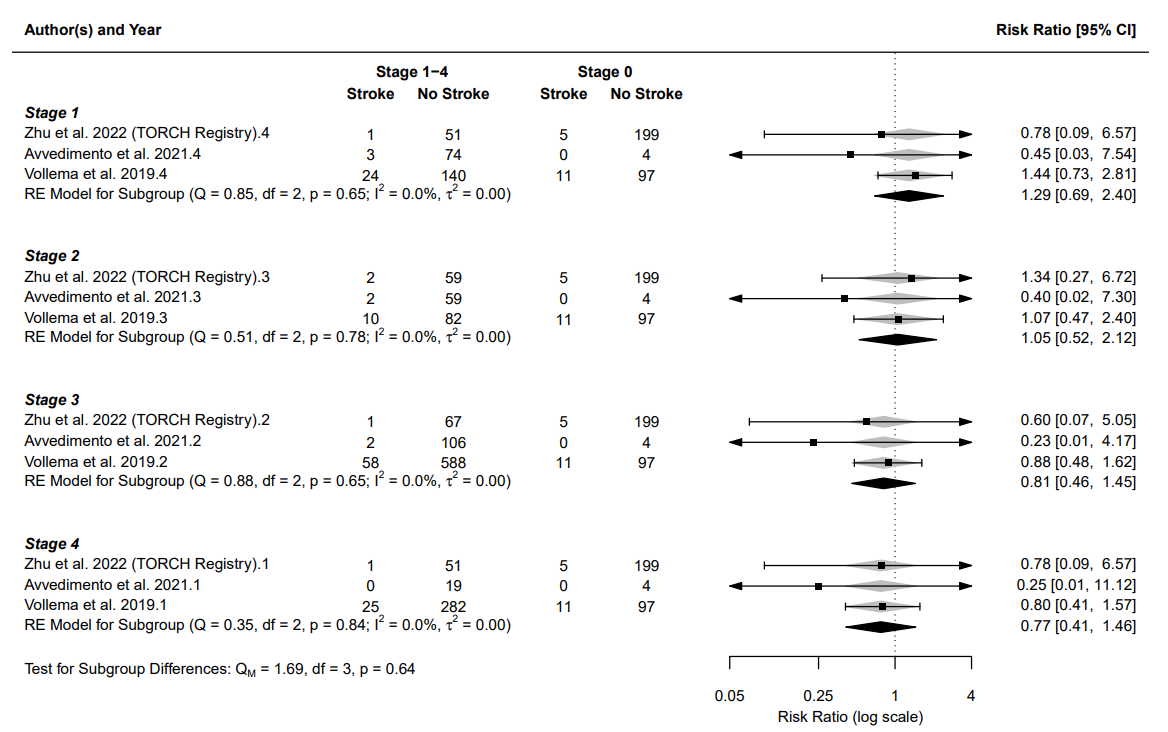


**Supplemental Methods**

**Study Selection and Eligibility Criteria.** Studies were excluded if they had inseparable cardiac damage staging or timing of AVR was undetermined following staging classification. No inclusion criterion was applied for the sample size.

**Staging classification for cardiac damage.** Of note, several studies^2–5^ incorporated additional variables in an attempt to provide a modified cardiac damage staging classification to the original classification by Généreux et al^6^; however, the pooled data in the current study from the aforementioned studies were only based on the initial staging classification.^6^

**Data extraction.** Given the limited data details in studies that report survival data because of aggregated summary statistics, conventional meta-analyses are prone to critical methodological issues.^7^ Hence, it is imperative to obtain individual patient data (IPD) from the original studies for an unbiased meta-analysis of survival data^8^; however, the challenges faced in obtaining original IPD owing to organizational, logistic, and privacy aspects make computational reconstruction of time-to-event data from published Kaplan-Meier curves more feasible for a meta-analysis of survival data.^9^ For data extraction and import of the quality data coordinates (time and survival probability) of the published Kaplan-Meier curves, an online digital software program WebPlotDigitizer version 4.6 was used. Coupled with the numbers at risk reported for several time points, the patient data of each included study were reconstructed using the R package IPDfromKM version 1.2.3.0.

For an additional layer of accuracy, the KM curves of each study cohort were computed. Two independent reviewers (O.M.A. and X.J.) assessed the computed Kaplan-Meier curves via visual inspection, and the authors of the original publications were contacted if further clarification was required.

**Aggregate data meta-analysis.** An aggregate data meta-analysis was performed for all studies that reported long-term outcomes (>12 months) for stroke in AS patients with cardiac staging at baseline (stage 0 vs stage 1-4). Relative risk (RR) with 95% CI and P values were calculated for each study and were then combined across the studies using a random-effects method (DerSimonian and Laird inverse variance).^10^ Forest plots were used to visualize the individual study and summary effect estimates.

**Statistical Analysis.** To summarize patients’ baseline characteristics and echocardiography parameters across the various cardiac damage stages, we performed a 1-group meta-analysis in a random effects model using the DerSimonian and Laird method. As a result, pooled estimates of baseline characteristics and echocardiography parameters were calculated as the inverse variance-weighted mean with 95% CIs. I² statistic describing the percentage of total variation across studies that is attributable to between-study heterogeneity rather than chance was calculated to assess the degree of statistical heterogeneity, and its accompanying P-value was obtained using the χ^2^ test.

**Results**

**Symptomatic severe AS and mortality and Stroke.**

For merged stages (stages 0-1, 2, and 3-4), at a median follow-up of 1.0 year (IQR, 0.6-2.0 y), a Kaplan-Meier curve analysis showed that patients with more advanced cardiac damage stages had significantly higher 1-year mortality event rates (log-rank χ^2^, 330.6; p<0.001).

In patients who had cardiac damage classified as stage 0-1, 1-year mortality was 7.5%. Whereas patients classified as stage 2 or stage 3-4 had a 1-year mortality of 10.6% and 19.3%, respectively (1-year HR in stage 2: 1.45 [95% CI, 1.23-1.71], p<0.001 and stage 3-4: 2.81 [95% CI, 2.40-3.29], p<0.001) (Central Illustration). At the final follow-up (5 years), all-cause mortality was 21.8%, 34.3%, and 52.5% in the patients with cardiac damage stage 0-1, 2, and 3-4, respectively (HR in stage 2: 1.48 [95% CI, 1.30-1.68], p <0.001 and stage 3-4: 2.79 [95% CI, 2.45-3.16], p <0.001, log-rank χ^2^, 465.6; p <0.001) (Figure 2). A significant incremental mortality increase with each cardiac damage stage was noted in patients with severe symptomatic AS (41.5% increase in mortality per each increment of stage [HR, 1.41; 95% CI, 1.35–1.48; p<0.001]).

Additionally, the relative risk of stroke between the different cardiac damage stages was assessed as an exploratory analysis. Only 3 studies reported data on stroke events stratified according to cardiac damage classification.^11–13^ The relative risk of stroke was not different between the cardiac damage stages in comparison to stage 0 (RR in stage 1: 1.29 [95% CI, 0.69-2.40], stage 2: 1.05 [95% CI, 0.52-2.12], Stage 3: 0.81 [95% CI, 0.46-1.45], and Stage 4: 0.77 [95% CI, 0.41-1.46] (Supplemental Figure 3).

**Asymptomatic moderate and severe AS and mortality.**

Data from individual stages 3 and 4 were not available in the originally published KM curves and were, thus, merged. Baseline characteristics stratified according to cardiac damage stage were available in 2 studies, which both reported complete data for each stage individually.

For merged stages, patients were stratified into 3 groups according to cardiac damage staging classification.^6^ A total of 4 studies (n=2217) (stage 0-1: n=949; stage 2: n=941; and stage 3-4: n=327) were pooled. Median follow-up was 4.1 years (IQR, 1.9-5.2 y). Mortality at 1 year increased in patients with higher cardiac damage at baseline. In patients who had cardiac damage classified as stage 0-1, 1-year mortality was 4.6%. (Supplemental Appendix)

Patients classified as stage 2 or stage 3-4 had a 1-year mortality of 9.2% and 17.6%, respectively (1-year HR in Stage 2: 1.93 [95% CI, 1.34-2.80]; p<0.001 and stage 3-4: 3.65 [95% CI, 2.44-5.46]; p<0.001) (Central Illustration). At the final follow-up (8 years), mortality was 30.1%, 51.7%, and 67.8% in the patients with cardiac damage stage 0-1, 2, and 3-4, respectively (HR in stage 2: 1.54 [95% CI, 1.29-1.84]; p<0.001 and stage 3-4: 2.71 [95% CI, 2.19-3.36]; p<0.001) (Figure 4).

**CV Mortality (Asymptomatic vs Symptomatic Cohorts)**

In total, 5 studies (3 symptomatic AS and 2 asymptomatic AS) reported stratified CV death during follow-up (n= 5101) (asymptomatic stage 0-1: n=261; stage 2: n=257; and stage 3-4: n=76; and symptomatic stage 0-1: n=885; stage 2: n=1831; and stage 3-4: n=1791). Similar to all-cause mortality, CV death was significantly different between the different groups (log-rank chi-square, 219.5; p<0.001); however, by comparing reciprocal cardiac stages from both cohorts (asymptomatic vs symptomatic), we found no significant difference in HRs for CV death across all stages at both 1-year and 4-year follow-up (Supplemental Table 5) (Supplemental Appendix) (Figure 6).

**References**

1. Higgins J, Morgan R, Rooney A, et al. ROBINS-E Development Group. Risk Of Bias In Non- randomized Studies - of Exposure (ROBINS-E). *Risk Bias Non-Randomized Stud - Expo ROBINS-E Launch Version* Accessed May 15, 2023. https://www.riskofbias.info/welcome/robins-e-tool.

2. Shamekhi J, Hasse C, Veulemans V, et al. A simplified cardiac damage staging predicts the outcome of patients undergoing TAVR—A multicenter analysis. *Catheter Cardiovasc Interv*. 2022;100:850–859.

3. Maeder MT, Weber L, Weilenmann D, et al. Invasive Hemodynamic Staging Classification of Cardiac Damage in Patients With Aortic Stenosis Undergoing Valve Replacement. *Can J Cardiol*. 2020;36:1667–1674.

4. Tastet L, Tribouilloy C, Maréchaux S, et al. Staging Cardiac Damage in Patients With Asymptomatic Aortic Valve Stenosis. *J Am Coll Cardiol*. 2019;74:550–563.

5. Berkovitch A, Barbash IM, Finkelstein A, et al. Validation of cardiac damage classification and addition of albumin in a large cohort of patients undergoing transcatheter aortic valve replacement. *Int J Cardiol*. 2020;304:23–28.

6. Généreux P, Pibarot P, Redfors B, et al. Staging classification of aortic stenosis based on the extent of cardiac damage. *Eur Heart J*. 2017;38:3351–3358.

7. Higgins JPT, Thomas J, Chandler J, Cumpston M, Li T, Page MJ WVA (editors). Cochrane Handbook for Systematic Reviews of Interventions version 6.2 (updated February 2021)

8. Lyman GH, Kuderer NM. The strengths and limitations of meta-analyses based on aggregate data. *BMC Med Res Methodol*. 2005;5:14.

9. Liu N, Zhou Y, Lee JJ. IPDfromKM: reconstruct individual patient data from published Kaplan-Meier survival curves. *BMC Med Res Methodol*. 2021;21:111.

10. DerSimonian R, Laird N. Meta-analysis in clinical trials. *Control Clin Trials*. 1986;7:177–188.

11. Vollema EM, Amanullah MR, Ng ACT, et al. Staging Cardiac Damage in Patients With Symptomatic Aortic Valve Stenosis. *J Am Coll Cardiol*. 2019;74:538–549.

12. Avvedimento M, Franzone A, Leone A, et al. Extent of Cardiac Damage and Mortality in Patients Undergoing Transcatheter Aortic Valve Implantation. *J Clin Med*. 2021;10:4563.

13. Zhu Q, Yuan Z, Xu Y, et al. Validation of a novel staging classification system based on the extent of cardiac damage among Chinese patients after transcatheter aortic valve replacement: A single‐center retrospective study. *Catheter Cardiovasc Interv*. 2022;99:1482–1489.
